# Astaxanthin Reverses Oxidative Stress‐Induced Dysfunction in Human Periodontal Ligament Stem Cells by Activating the Nrf2/ARE Pathway

**DOI:** 10.1155/sci/1662288

**Published:** 2026-01-21

**Authors:** Jingwen Chi, Xiaofei Yu, Hui Zhang, Mengyu Jiao, Peiyan Wang, Kexin Wang, Tianlu Wang, Jing Deng

**Affiliations:** ^1^ Department of Stomatology, The Affiliated Hospital of Qingdao University, Qingdao, 266003, China, qdu.edu.cn; ^2^ School of Stomatology, Qingdao University, Qingdao, 266003, China, qdu.edu.cn; ^3^ Dental Digital Medicine and 3D Printing Engineering Laboratory of Qingdao, Qingdao University, Qingdao, 266003, Shandong, China, qdu.edu.cn

**Keywords:** astaxanthin, human periodontal ligament stem cells, Nrf2/ARE, oxidative stress, periodontitis

## Abstract

**Background:**

Oxidative stress plays a crucial role in the pathogenesis of periodontitis and compromises the regenerative potential of human periodontal ligament stem cells (hPDLSCs). Astaxanthin (ASX), a potent natural antioxidant with both lipophilic and hydrophilic properties, has been shown to scavenge reactive oxygen species (ROS). However, its protective effects on hPDLSCs under oxidative stress remain largely unexplored.

**Methods:**

hPDLSCs were isolated and characterized. An oxidative stress model was established by exposing cells to 300 μM H_2_O_2_ for 6 h, followed by treatment with 10 μM ASX. Cellular viability, cytoskeletal integrity, ROS accumulation, inflammatory cytokine expression, osteogenic differentiation, and activation of the Nrf2/ARE pathway were assessed.

**Results:**

ASX significantly reduced intracellular and mitochondrial ROS levels, preserved mitochondrial membrane potential, and inhibited H_2_O_2_‐induced expression of TNF‐*α*, IL‐1*β*, IL‐6, and MCP‐1. Moreover, ASX promoted osteogenic differentiation, as evidenced by enhanced alkaline phosphatase (ALP) activity, increased mineralized nodule formation, and upregulation of RUNX2, OCN, and COL1. Mechanistically, ASX activated the Nrf2/ARE pathway, leading to increased expression of Nrf2 and its downstream antioxidant enzymes (HO‐1, NQO‐1, and GCLC).

**Conclusion:**

These findings demonstrate that ASX ameliorates oxidative stress‐induced injury in hPDLSCs via the Nrf2/ARE signaling pathway, exerting antioxidative, anti‐inflammatory, and pro‐osteogenic effects. This suggests its therapeutic potential for promoting periodontal regeneration under oxidative microenvironments.

## 1. Introduction

Periodontitis is a chronic inflammatory disease characterized by the progressive destruction of periodontal supporting tissues [1]. It is a leading cause of tooth loss in adults and is associated with various systemic conditions [2]. Its pathogenesis involves dysbiosis of the oral microbiota and excessive activation of the host immune system, with oxidative stress recognized as a key driver of disease progression [3–5]. During periodontitis, excessive reactive oxygen species (ROS) disrupt cellular redox homeostasis, leading to oxidative damage to DNA, proteins, and lipids, and activating multiple stress‐ and inflammation‐related signaling pathways [6]. These changes ultimately induce apoptosis and impair tissue repair. Increasing evidence suggests that oxidative stress not only accelerates periodontal tissue destruction but also diminishes the regenerative potential of local progenitor and stem cells, including human periodontal ligament stem cells (hPDLSCs), thereby limiting periodontal regeneration [7].

hPDLSCs are mesenchymal stem cells residing within the periodontal ligament, possessing self‐renewal capacity and multidirectional differentiation potential [8]. They play a pivotal role in differentiating into osteoblast‐ and cementoblast‐like cells, maintaining periodontal homeostasis, and mediating post‐injury regeneration. However, under oxidative stress, hPDLSCs exhibit reduced viability, mitochondrial dysfunction, and impaired osteogenic differentiation. Therefore, strategies aimed at mitigating oxidative damage are critical for restoring hPDLSC function and preventing periodontal tissue destruction [9].

Astaxanthin (ASX) is a xanthophyll carotenoid found in marine organisms such as microalgae, krill, and salmon. Its molecular structure, characterized by conjugated double bonds and polar end groups, confers strong membrane affinity and potent antioxidant activity [10]. ASX has a markedly higher ROS‐scavenging efficiency than vitamins C and E, and functions in both lipid and aqueous environments to neutralize free radicals and inhibit lipid peroxidation [11]. It has demonstrated anti‐inflammatory, antiapoptotic, and cytoprotective effects in various oxidative stress models [12]. However, its protective role in hPDLSCs under oxidative stress and the underlying mechanisms remain unclear.

The nuclear factor erythroid 2–related factor 2 (Nrf2)/antioxidant response element (ARE) pathway is one of the principal cellular defense mechanisms against oxidative stress [13]. Upon activation, Nrf2 translocates into the nucleus, binds to AREs, and upregulates a battery of antioxidant and cytoprotective genes, including heme oxygenase‐1 (HO‐1), NAD(P)H quinone oxidoreductase 1 (NQO1), and glutamate–cysteine ligase catalytic (GCLC) subunit, thereby attenuating oxidative injury and maintaining cellular homeostasis [14]. Activation of the Nrf2/ARE pathway has been shown to alleviate oxidative stress and improve stem cell function [15]. Given ASX’s redox‐regulating properties, its cytoprotective effects may be closely related to Nrf2/ARE pathway activation.

Based on these considerations, the present study established an H_2_O_2_‐induced oxidative stress model in hPDLSCs to investigate the protective effects of ASX and to explore the potential involvement of the Nrf2/ARE signaling pathway, providing a theoretical basis for clinical strategies aimed at mitigating periodontal tissue destruction.

## 2. Materials and Methods

### 2.1. Cell Isolation and Culture

hPDLSCs were obtained from healthy premolars extracted for orthodontic reasons from donors aged 12–18 years, after obtaining written informed consent from each participant or their legal guardian. Periodontal ligament tissue was gently scraped from the middle third of the tooth root and digested in a solution containing 3 mg/mL type I collagenase and 4 mg/mL dispase at 37°C for 60 min. The resulting cell suspension was filtered through a 70 μm cell strainer (BD Falcon, USA) and seeded in *α*‐MEM supplemented with 10% fetal bovine serum (FBS) and 1% (v/v) penicillin–streptomycin. Cells were maintained at 37°C in a humidified atmosphere containing 5% CO_2_, with the culture medium replaced every 2–3 days. Cells at passages 3–5 were used for all experiments [16].

### 2.2. Flow Cytometry Characterization

To verify their mesenchymal stem cell phenotype, hPDLSCs were digested with 0.25% trypsin–EDTA, resuspended in PBS, and adjusted to 1 × 10^6^ cells per tube. Cells were incubated with fluorescein isothiocyanate (FITC)‐conjugated monoclonal antibodies against CD44, CD90, CD105, CD34, and CD45 for 30 min at 4°C in the dark, with isotype‐matched antibodies used as controls. Samples were analyzed using a BD FACSCanto II flow cytometer (BD Biosciences, USA), and data were processed with FlowJo v10.8.1 (BD, USA) [17].

### 2.3. Multilineage Differentiation Assays

#### 2.3.1. Osteogenic Differentiation

Cells were cultured in *α*‐MEM containing 10% FBS, 50 μg/mL ascorbic acid, 10 mM *β*‐glycerophosphate, and 100 nM dexamethasone for 14 days, with medium changes every 1 day. ALP activity was assessed using a BCIP/NBT ALP staining kit (Beyotime, China). After 21 days, mineralized nodules were stained with 0.1% Alizarin Red S (ARS; Sigma–Aldrich, USA), eluted with 10% cetylpyridinium chloride (Sigma–Aldrich, USA), and quantified spectrophotometrically at 562 nm (SpectraMax iD3, Molecular Devices, USA).

#### 2.3.2. Adipogenic differentiation

Cells were cultured in *α*‐MEM containing 10% FBS, 1 μM dexamethasone, 0.5 mM isobutylmethylxanthine, 10 μg/mL insulin, and 200 μM indomethacin for 21 days and stained with 0.5% Oil Red O.

### 2.4. Oxidative Stress Model and ASX Treatment

Hydrogen peroxide (H_2_O_2_; Sigma–Aldrich, USA) was freshly diluted in serum‐free medium and applied at different concentrations (0, 100, 200, 300, 400, and 500 μM) for 1, 3, 6, or 12 h. Cell viability was assessed using the CCK‐8 assay, and the optimal oxidative condition (300 μM H_2_O_2_ for 6 h) was chosen for subsequent experiments. ASX (Sigma–Aldrich, USA; purity ≥ 97%) was dissolved in dimethyl sulfoxide (DMSO) to prepare a 10 mM stock solution, stored at −20°C in the dark, and diluted to the desired concentration immediately before use (final DMSO ≤ 0.1%). After oxidative stress induction with H_2_O_2_, cells were treated with 10 μM ASX for 24 h to evaluate its protective effects.

### 2.5. Cell Viability Assay (CCK‐8)

Cells were seeded into 96‐well plates at a density of 5 × 10^3^ cells/well and treated according to the experimental design. Ten microliters of CCK‐8 reagent were added to each well, and the cells were incubated at 37°C for 2 h. Absorbance was measured at 450 nm using a SpectraMax iD3 microplate reader, and results were normalized to the control group.

### 2.6. siRNA Transfection

Cells were transiently transfected with an siRNA targeting human NFE2L2 (Nrf2) (siNrf2; RiboBio, Guangzhou, China) or a nontargeting negative control (siNC; RiboBio) using a RiboBio transfection reagent according to the manufacturer’s instructions. Cells at 40%–60% confluence were transfected in antibiotic‐free medium with a final siRNA concentration of 20 nM; after 6–8 h, the medium was replaced with complete *α*‐MEM (10% FBS), and cultures were maintained under standard conditions. Knockdown efficiency was verified by qPCR (24–48 h) and Western blot (48–72 h) post‐transfection; only experiments achieving ≥60% reduction of Nrf2 mRNA/protein were included in the analysis.

### 2.7. Cytoskeletal Staining

Cells grown on glass coverslips were fixed with 4% paraformaldehyde (Beyotime, China) for 15 min, permeabilized with 0.1% Triton X‐100 (Sigma–Aldrich, USA) for 5 min, and blocked with 1% bovine serum albumin (BSA; Sigma–Aldrich, USA) for 30 min. F‐actin was stained with TRITC–phalloidin (Sigma–Aldrich, USA) for 30 min, and nuclei were counterstained with DAPI (Beyotime, China). Images were acquired using a fluorescence microscope.

### 2.8. ROS and Mitochondrial Function Assays

Total intracellular ROS levels were measured using 10 μM 2′,7′‐dichlorodihydrofluorescein diacetate (DCFH‐DA; Beyotime, China) at 37°C for 30 min in the dark, followed by three washes with serum‐free medium to remove excess probe. Mitochondrial ROS were detected using 5 μM MitoSOX Red (Invitrogen, USA) at 37°C for 20 min in the dark, followed by three washes.

Mitochondrial membrane potential (*ΔΨ*m) was assessed with a JC‐1 assay kit (Beyotime, China). Cells were incubated with JC‐1 working solution at 37°C for 20 min, washed twice with JC‐1 buffer, and imaged. The red/green fluorescence intensity ratio was used as an indicator of *ΔΨ*m. For all imaging, exposure settings were kept constant, and fluorescence intensity was quantified using ImageJ v1.53k (NIH, USA).

### 2.9. Immunofluorescence (IF) for Inflammatory Markers

IF for TNF‐*α* and IL‐1*β* was performed as follows: cells were fixed with 4% paraformaldehyde for 15 min, permeabilized with 0.1% Triton X‐100 for 5 min, and blocked with 1% BSA for 30 min at room temperature. Primary antibodies against TNF‐*α* and IL‐1*β*(Beyotime, China) were applied overnight at 4°C, followed by species‐matched fluorophore‐conjugated secondary antibodies for 1 h at room temperature; nuclei were counterstained with DAPI. Images were acquired with identical exposure/gain across groups (scale bar, 200 μm). Fluorescence was quantified in ImageJ v1.53k (NIH, USA) as single‐cell mean fluorescence intensity (MFI) after background subtraction using DAPI‐seeded watershed segmentation.

### 2.10. Quantitative Real‐Time PCR (qPCR)

Total RNA was extracted using the RNA isolator reagent (Vazyme, China) and reverse‐transcribed with the HiScript III All‐in‐one RT SuperMix (Vazyme, China). qPCR was performed using Taq Pro Universal SYBR qPCR Master Mix (Vazyme, China) on a QuantStudio 5 Real‐Time PCR System (Applied Biosystems, USA). GAPDH was used as the internal control, and relative gene expression levels were calculated using the 2^−*ΔΔ*Ct^ method. Primer sequences are listed in Table 1.

**Table 1:** Primer sequences.

Gene	Forward primer (5′→3′)	Reverse primer (5′→3′)
*GAPDH*	TGACTTCAACAGCGACACCCA	CACCCTGTTGCTGTAGCCAAA
*TNF-β*	CGAGTCTGGGCAGGTCTA	AGGGTGTCTGAAGGAGGG
*IL-1β*	ACAGTGGCAATGAGGATG	TGTAGTGGTGGTCGGAGA
*IL-6*	GGAGACTTGCCTGGTGAA	GCATTTGTGGTTGGGTCA
*MCP-1*	CAGAAGTGGGTTCAGGAT	TTGGGTTGTGGAGTGAGTGT
*RUNX2*	TACCTGAGCCAGATGACG	ATGAAATGCTTGGGAACT
*OCN*	AGGGCAGCGAGGTAGTGA	CCTGAAAGCCGATGTGGT
*ALP*	ACGGTCACTCTGTATCCC	AAAGCCATGAATGTCTTCT
*COL1*	CGAAGACATCCCACCAATC	ATCACGTCATCGCACAACA
*Nrf2*	TGTAAGTCCTGGTCATCG	TTGCCCTAAGTTCATCTC
*HO-1*	CTCTTGGCTGGCTTCCTT	GGCTCCTTCCTCCTTTCC
*NQO1*	ACCTCCTTTACCAGATGC	GTGAAGATGAAGGCAACA
*GCLC*	ATGGGAGTTACATGATTGA	TTGTTAAGGTACTGGGAAA

### 2.11. Western Blot Analysis

Total protein was extracted using RIPA lysis buffer (Beyotime, China) and quantified with a bicinchoninic acid (BCA) assay (Thermo, USA). Equal amounts of protein (20–30 μg) were separated by 10% SDS–PAGE and transferred onto polyvinylidene difluoride (PVDF) membranes (Millipore, USA). Membranes were blocked with 5% nonfat milk for 1 h at room temperature and incubated overnight at 4°C with primary antibodies against TNF‐*α*, IL‐1*β*, IL‐6, MCP‐1, RUNX2, OCN, ALP, COL1, Nrf2, HO‐1, NQO1, GCLC, and GAPDH (Proteintech, BIOSS, or Abcam; all at 1:1000 dilution, with catalog numbers available upon request). After washing, membranes were incubated with horseradish peroxidase (HRP)‐conjugated secondary antibodies (Proteintech, China; 1:5000) for 1 h at room temperature. Protein bands were visualized using an enhanced chemiluminescence (ECL) detection system (Thermo, USA) and analyzed by densitometry using ImageJ software (NIH, USA).

### 2.12. Statistical Analysis

All statistical analyses were performed using GraphPad Prism 9.0 software. Data are presented as the mean ± standard deviation (SD), with a sample size of *n* ≥ 3. Normality and homogeneity of variance were verified before statistical testing. Differences among groups were assessed using one‐way analysis of variance followed by Tukey’s post hoc test. A *p*‐value < 0.05 was considered statistically significant.

## 3. Results

### 3.1. Identification of hPDLSCs

The immunophenotype of isolated hPDLSCs was analyzed by flow cytometry (Figure 1A). The cells exhibited high expression of mesenchymal stem cell markers CD44 (99.5%), CD90 (99.6%), and CD105 (99.7%), whereas the hematopoietic markers CD34 (1.68%) and CD45 (2.25%) were expressed at very low levels. This profile was consistent with the immunophenotypic characteristics of mesenchymal stem cells. After 14 days of osteogenic induction, alkaline phosphatase (ALP) staining showed strong enzymatic activity (Figure 1B), and ARS staining revealed abundant calcium deposition and mineralized nodules (Figure 1C), confirming their osteogenic capacity. Following 21 days of adipogenic induction, Oil Red O staining detected abundant intracellular lipid droplets (Figure 1D), demonstrating their multilineage differentiation potential.

Figure 1Identification of human periodontal ligament stem cells (hPDLSCs). (A) Flow cytometric analysis of mesenchymal stromal cell surface markers in hPDLSCs (the panel shows the gating strategy and marker distributions). (B–D) Representative staining demonstrating multilineage potential: ALP staining (early osteogenesis), Alizarin Red S (ARS) staining for mineralization (late osteogenesis), and Oil Red O staining for adipogenesis. Images were acquired under identical exposure/gain; scale bars, 200 μm.

**Figure:**
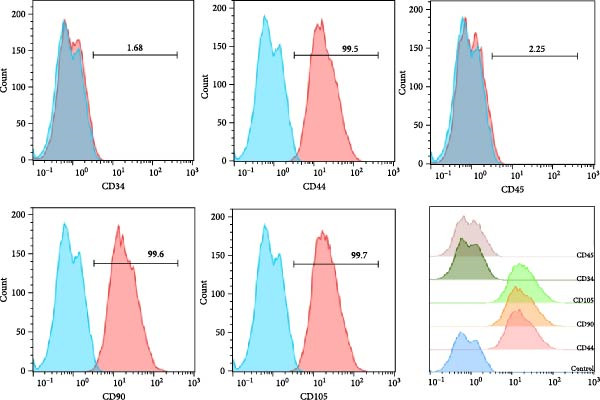
(A)

**Figure:**
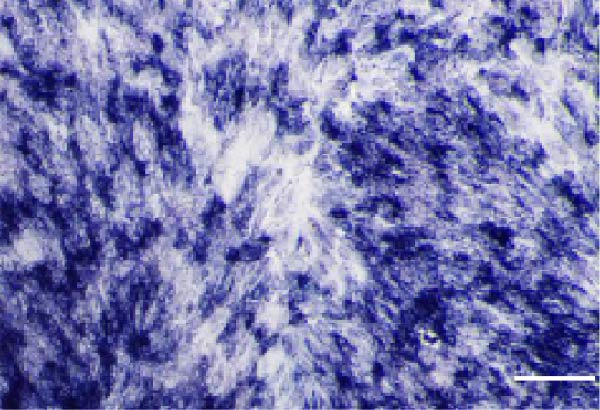
(B)

**Figure:**
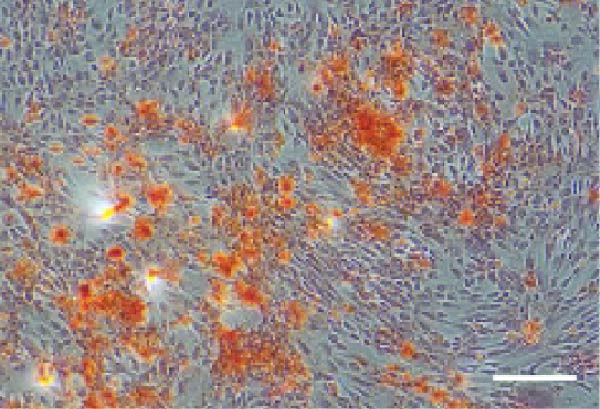
(C)

**Figure:**
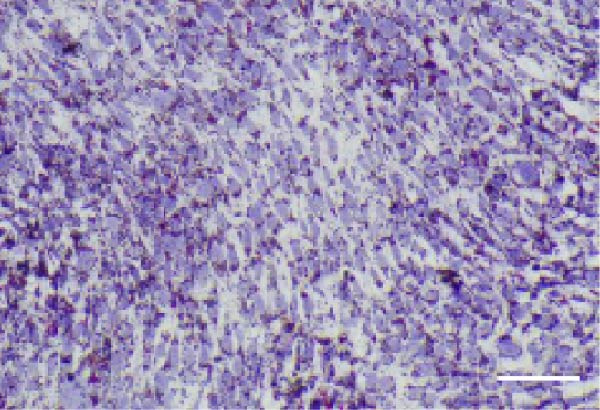
(D)

### 3.2. Establishment of the Oxidative Stress Model and Selection of ASX Concentration

To establish an in vitro oxidative stress model, hPDLSCs were treated with different concentrations of H_2_O_2_ (0, 100, 200, 300, 400, and 500 μM) for varying durations (1, 3, 6, and 12 h). Cell viability, assessed by the CCK‐8 assay, declined in both a concentration‐ and time‐dependent manner. Among the tested conditions, 300 μM H_2_O_2_ for 6 h reduced viability to ~65% of the control, producing substantial oxidative injury while retaining an adequate number of living cells for subsequent experiments. This condition was therefore selected as the optimal oxidative stress induction protocol (Figure 2A–D).

Figure 2Effects of H_2_O_2_ and astaxanthin (ASX) on hPDLSCs. (A–D) CCK‐8 viability of PDLSCs after H_2_O_2_ exposure (0, 100, 200, 300, 400, 500 μM) for 1 h (A), 3 h (B), 6 h (C), and 12 h (D). (E) Chemical structure of astaxanthin. (F) CCK‐8 viability after ASX treatment (0–16 μM, 24 h; DMSO vehicle). (G) Representative phalloidin/DAPI images for Control, H_2_O_2_, ASX, and H_2_O_2_ + ASX (F‐actin, phalloidin/red; nuclei, DAPI/blue); identical exposure/gain; scale bar, 200 μm. (H,I) Single‐cell quantification of cell spread area (H) and mean F‐actin intensity (I). Values were normalized to control ( = 100%). Data are presented as mean ± SD (*n* = 3). Statistical significance:  ^∗^ *p* < 0.05,  ^∗∗^ *p* < 0.01,  ^∗∗∗^ *p* < 0.001,  ^∗∗∗∗^ *p* < 0.0001.

**Figure:**
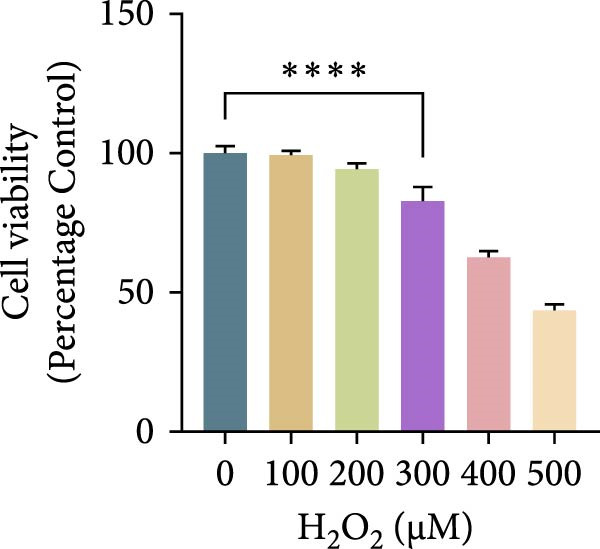
(A)

**Figure:**
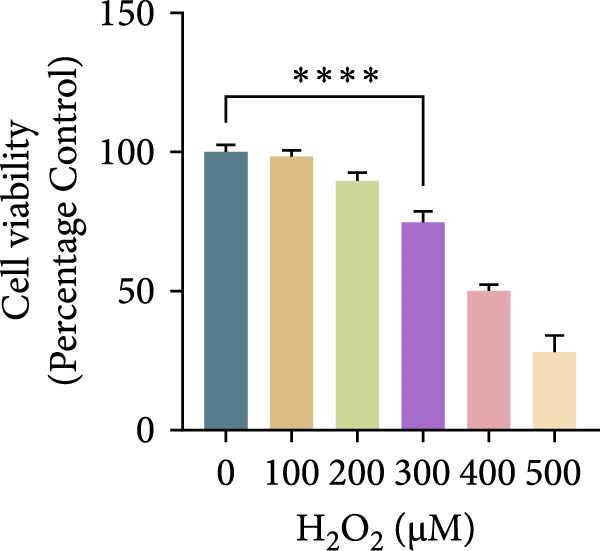
(B)

**Figure:**
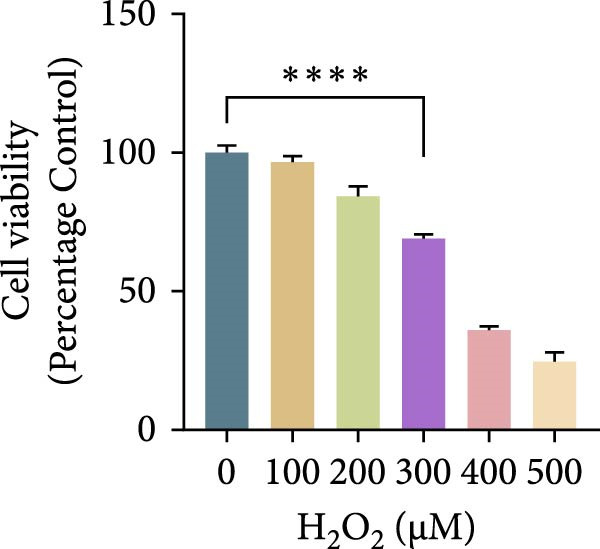
(C)

**Figure:**
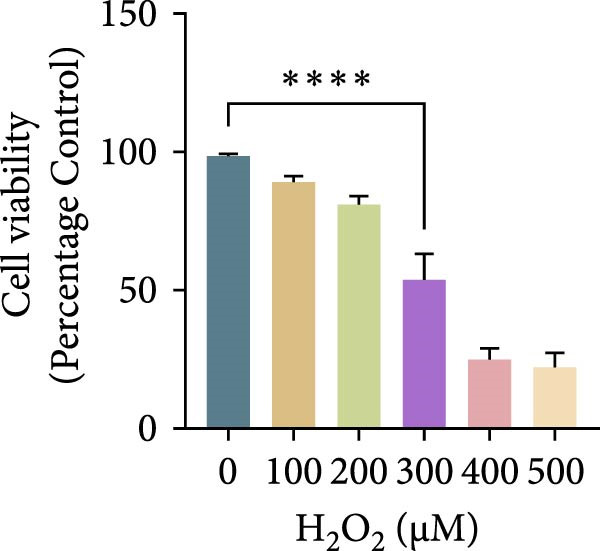
(D)

**Figure:**
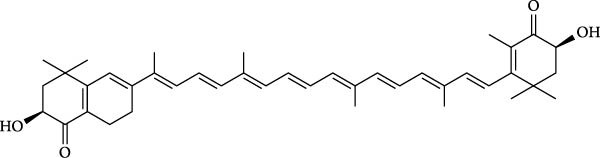
(E)

**Figure:**
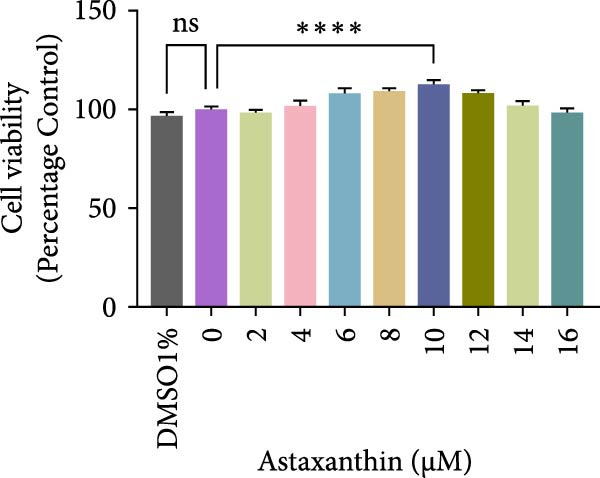
(F)

**Figure:**
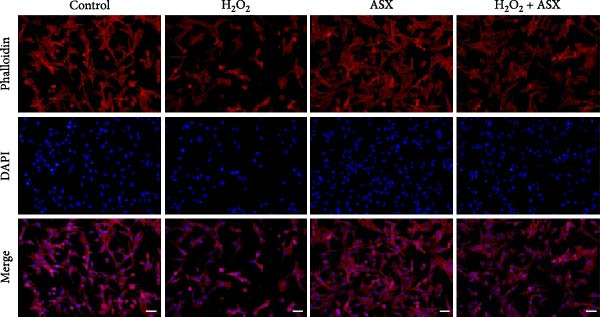
(G)

**Figure:**
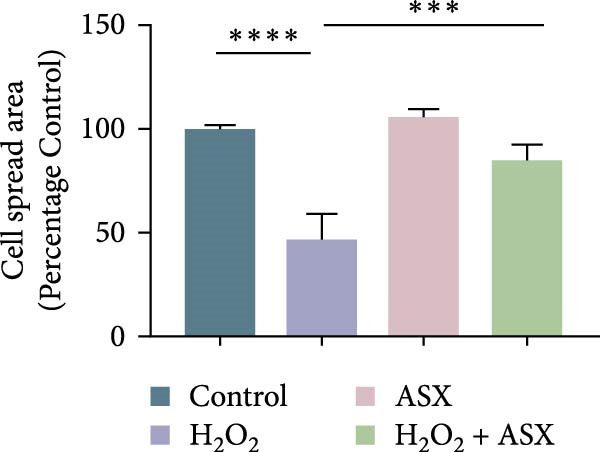
(H)

**Figure:**
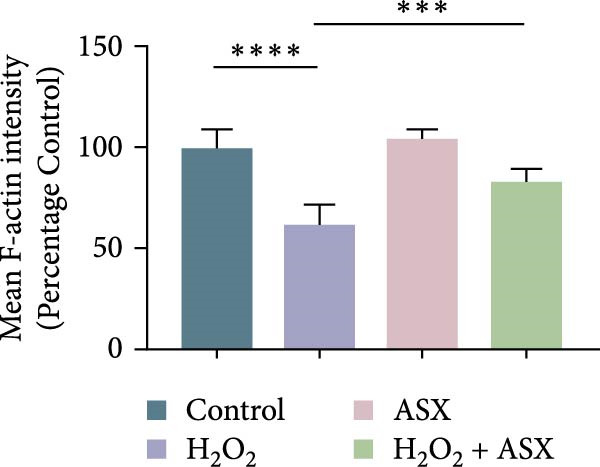
(I)

The safety profile of ASX was evaluated by dissolving the compound in DMSO and preparing a concentration gradient (2, 4, 8, 10, and 16 μM). Following 24 h of treatment, CCK‐8 assays indicated that ASX up to 16 μM did not significantly affect cell viability compared with controls. Considering both biosafety and biological efficacy, 10 μM ASX was chosen for further experiments (Figure 2F).

Morphological changes were assessed by phalloidin staining. H_2_O_2_ exposure markedly disrupted the cytoskeleton, characterized by reduced F‐actin density, shortened stress fibers, and cell shrinkage. In contrast, cells treated with ASX under oxidative stress retained relatively intact cytoskeletal organization, exhibiting continuous stress fibers and a well‐spread morphology comparable to untreated controls (Figure 2G). Quantification of cell spread area and F‐actin intensity showed a significant reduction in the H_2_O_2_ group, which was partially restored by ASX (Figure 2H,I).

### 3.3. ASX Attenuates ROS Accumulation and Preserves Mitochondrial Function

To assess the ability of ASX to counteract oxidative stress, hPDLSCs were first exposed to H_2_O_2_ to induce oxidative injury, followed by treatment with ASX. DCFH‐DA staining revealed that H_2_O_2_ stimulation significantly increased intracellular ROS fluorescence intensity compared with the control group (*p* < 0.01), indicating a marked elevation in oxidative burden (Figure 3A). Similarly, MitoSOX Red staining demonstrated significantly higher mitochondrial ROS levels in H_2_O_2_‐treated cells (*p* < 0.01), suggesting that mitochondria represent a major source of ROS under oxidative challenge (Figure 3B).

Figure 3Effects of ASX on intracellular ROS levels and mitochondrial membrane potential in hPDLSCs under oxidative stress. (A) Intracellular ROS detected by DCFH‐DA in control, H_2_O_2_, ASX, and H_2_O_2_ + ASX. (B) Mitochondrial ROS detected by MitoSOX Red. (C) JC‐1 for mitochondrial membrane potential (*ΔΨ*m; red = J‐aggregates/high *ΔΨ*m; green = J‐monomers/low *ΔΨ*m). Representative images acquired with identical exposure/gain; scale bar, 200 μm. (D–F) Single‐cell quantification for (A–C) (Fiji background subtraction; DAPI‐seeded watershed for per‐cell ROIs); values normalized to control ( = 100%). Data are presented as mean ± SD (*n* = 3). Statistical significance:  ^∗^ *p* < 0.05,  ^∗∗^ *p* < 0.01,  ^∗∗∗^ *p* < 0.001,  ^∗∗∗∗^ *p* < 0.0001.

**Figure:**
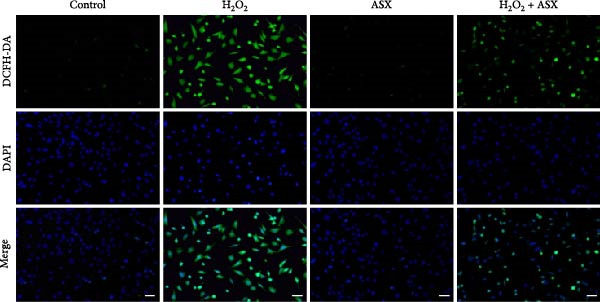
(A)

**Figure:**
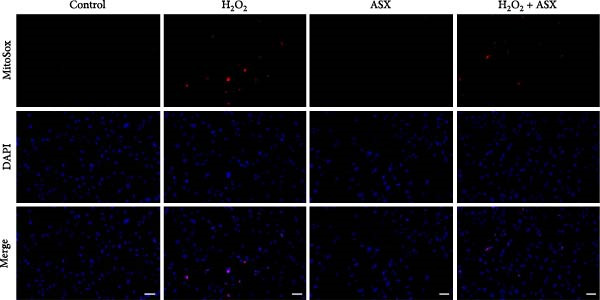
(B)

**Figure:**
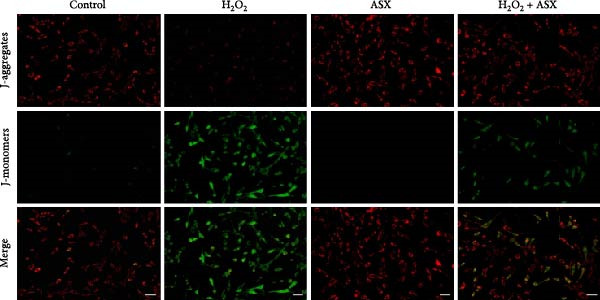
(C)

**Figure:**
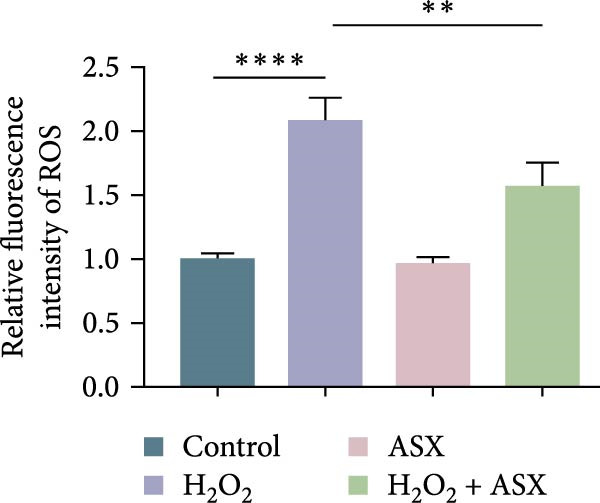
(D)

**Figure:**
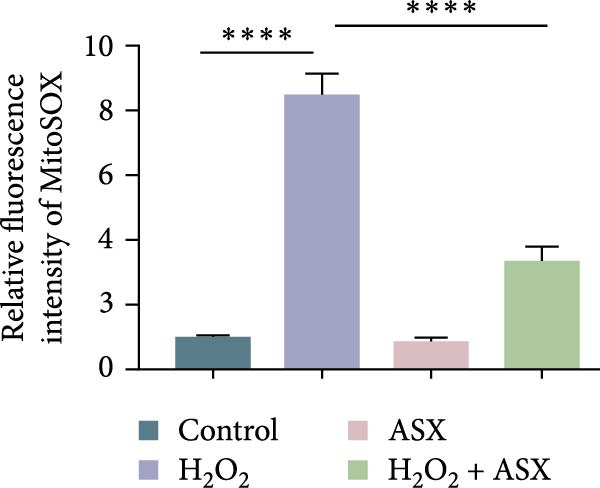
(E)

**Figure:**
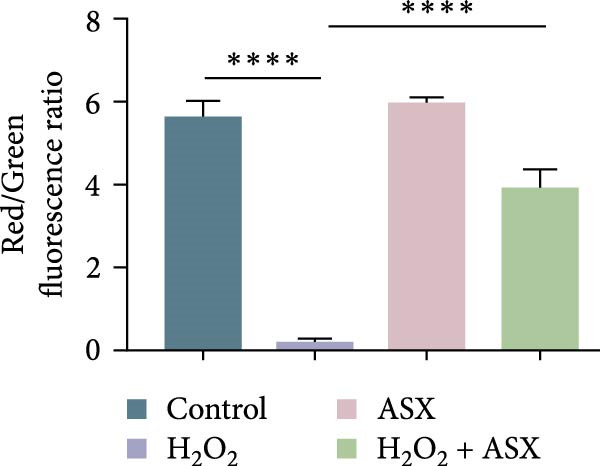
(F)

Mitochondrial function was further examined by JC‐1 staining. In control cells, JC‐1 aggregated within polarized mitochondria, producing strong red fluorescence. After H_2_O_2_ exposure, red fluorescence was markedly decreased with a concomitant increase in green fluorescence, reflecting mitochondrial depolarization and dysfunction (*p* < 0.01) (Figure 3C).

Subsequent treatment with 10 μM ASX significantly reduced both intracellular and mitochondrial ROS levels compared with the H_2_O_2_ group (*p* < 0.05) and partially restored *ΔΨ*m toward normal levels (*p* < 0.05). Morphological assessment further confirmed that ASX‐treated cells exhibited a more preserved mitochondrial network with reduced swelling and less structural disruption.

Collectively, these findings indicate that ASX, when administered after oxidative stress induction, effectively mitigates ROS accumulation and alleviates mitochondrial dysfunction, thereby supporting mitochondrial integrity and maintaining cellular energy homeostasis in hPDLSCs.

### 3.4. ASX Suppresses Oxidative Stress‐Induced Inflammatory Response

To determine whether ASX modulates inflammation under oxidative stress, hPDLSCs were exposed to 300 μM H_2_O_2_ for 6 h followed by 10 μM ASX. IF showed that H_2_O_2_ markedly increased TNF‐*α* and IL‐1*β* signals (*p* < 0.01), as shown in Figure 4A,B (single‐cell MFI quantified in the right panels). In line with the imaging data, qPCR revealed significant transcriptional upregulation of TNF‐*α*, IL‐1*β*, IL‐6, and MCP‐1 (*p* < 0.001; Figure 4C–F), and Western blot confirmed elevated protein levels (*p* < 0.001; Figure 4G, densitometry in Figure 4H–K).

Figure 4Effects of ASX on the expression of inflammatory cytokines in hPDLSCs under oxidative stress. (A) IF of TNF‐*α* with DAPI counterstain and single‐cell MFI quantification. (B) IF of IL‐1*β* and single‐cell MFI quantification. All IF images acquired under identical exposure/gain; scale bar, 200 μm. (C–F) qPCR of TNF‐*α*, IL‐1*β*, IL‐6, MCP‐1, normalized to GAPDH and to control. (G) Representative Western blots for these proteins; GAPDH served as loading control. (H–K) Densitometric quantification of Western blots normalized to GAPDH and to Control.  ^∗∗^Values were normalized to control ( = 100%). Data are presented as mean ± SD (*n* = 3). Statistical significance:  ^∗^ *p* < 0.05,  ^∗∗^ *p* < 0.01,  ^∗∗∗^ *p* < 0.001,  ^∗∗∗∗^ *p* < 0.0001.

**Figure:**
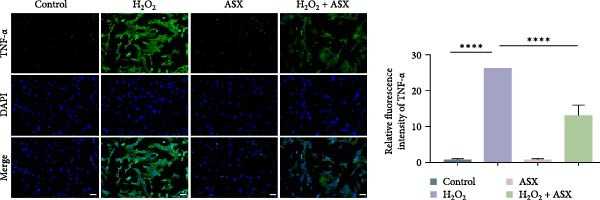
(A)

**Figure:**
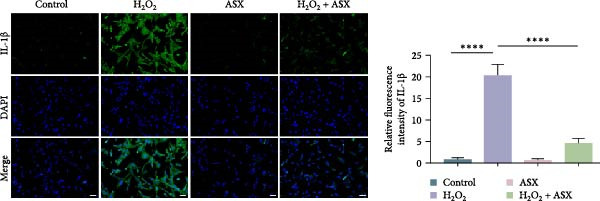
(B)

**Figure:**
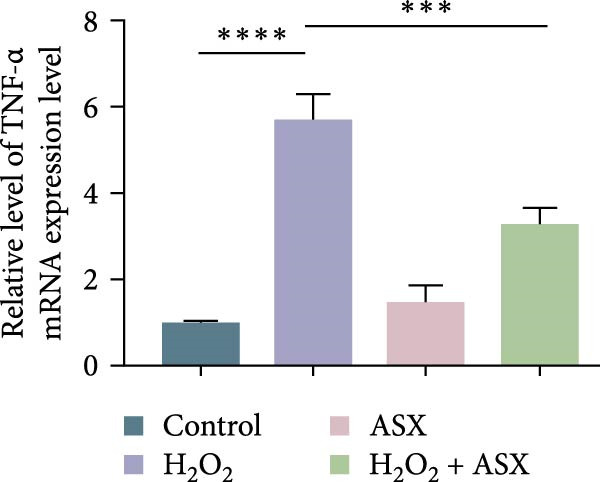
(C)

**Figure:**
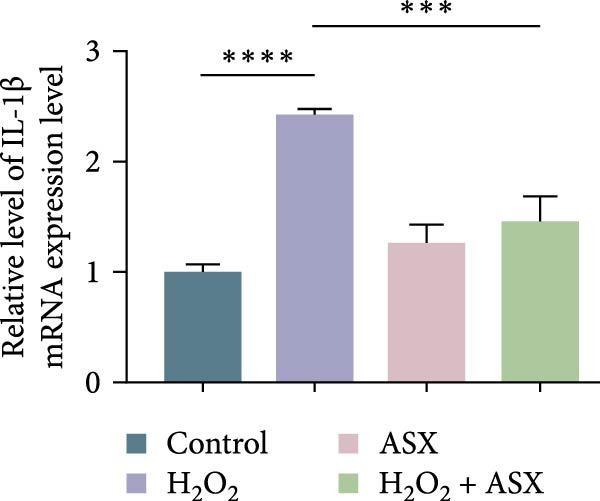
(D)

**Figure:**
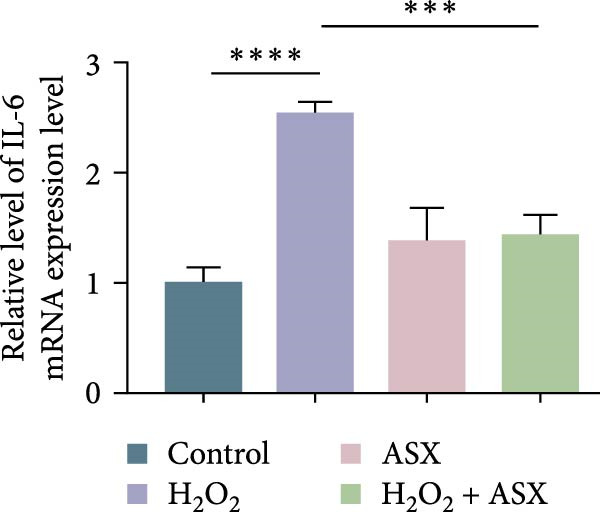
(E)

**Figure:**
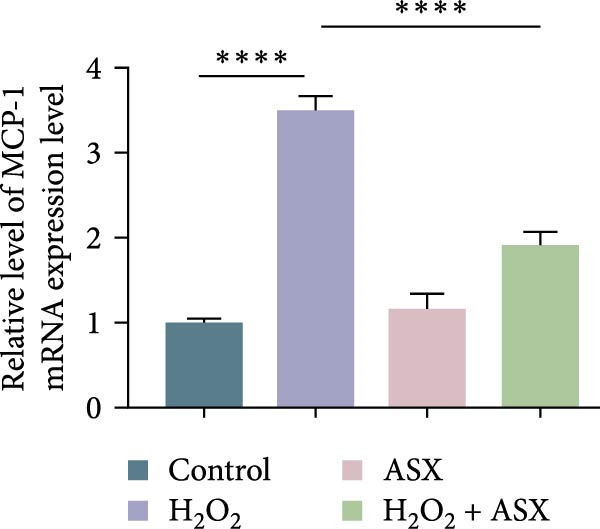
(F)

**Figure:**
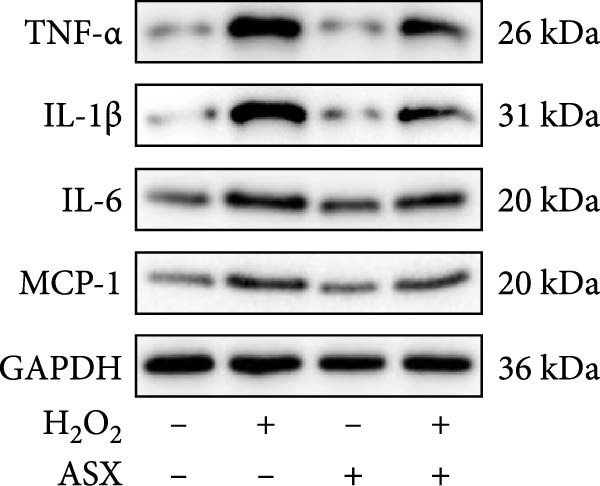
(G)

**Figure:**
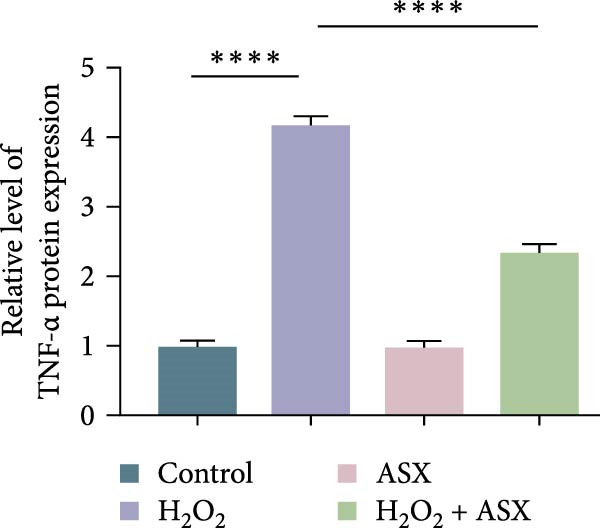
(H)

**Figure:**
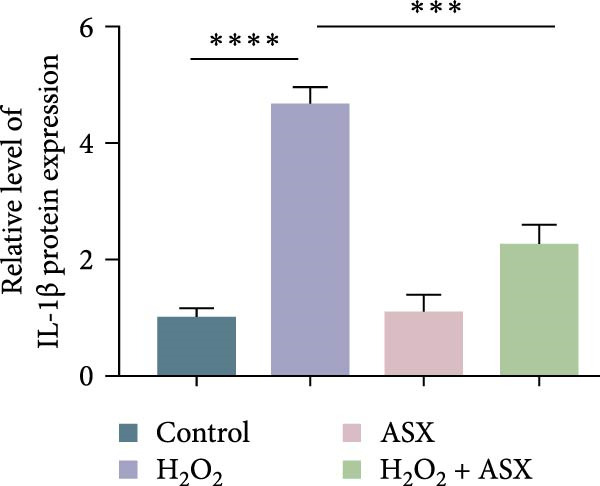
(I)

**Figure:**
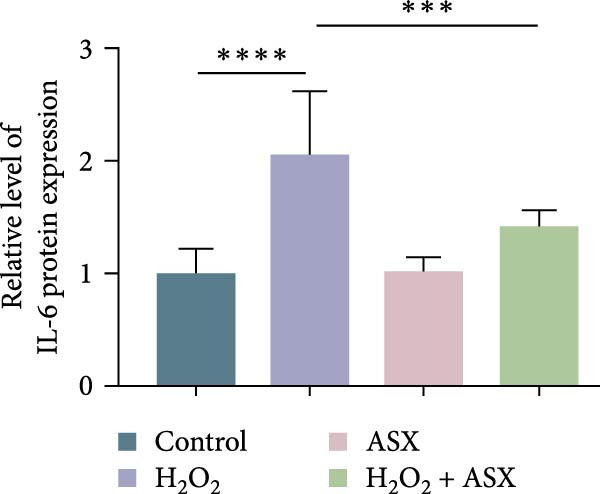
(J)

**Figure:**
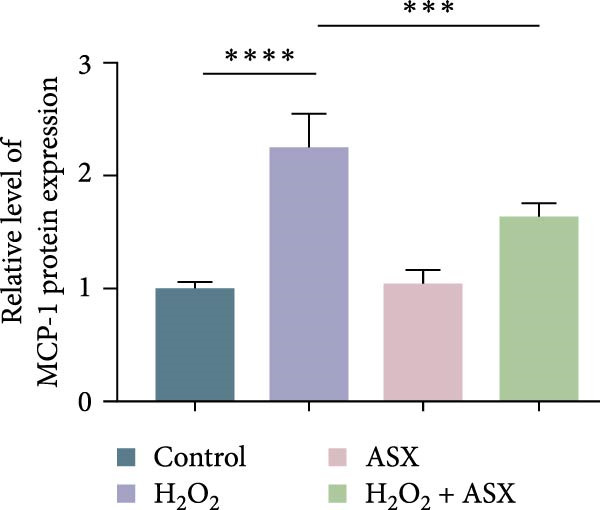
(K)

Subsequent ASX treatment significantly attenuated these inflammatory readouts, reducing IF MFI, mRNA, and protein levels toward control (*p* < 0.01), as depicted in Figure 4A–K. These results indicate that ASX effectively suppresses the H_2_O_2_‐triggered pro‐inflammatory response in hPDLSCs.

### 3.5. ASX Restores Osteogenic Differentiation Capacity After Oxidative Stress

We next evaluated whether ASX could reverse the impairment of osteogenic differentiation caused by oxidative stress. Early osteogenesis was assessed by ALP staining and quantitative analysis. H_2_O_2_ treatment significantly reduced ALP activity (*p* < 0.01 vs. control), indicating compromised early osteogenic potential. Late‐stage mineralization was also markedly inhibited, as demonstrated by ARS staining and quantification, which showed decreased calcified nodules and mineral deposition (*p* < 0.01). Treatment with ASX after H_2_O_2_ exposure significantly improved both parameters, with ALP activity and ARS‐measured mineralization levels increased compared with the H_2_O_2_ group (*p* < 0.05, Figure 5A–D).

Figure 5Effects of ASX on osteogenic differentiation of hPDLSCs under oxidative stress. (A) Representative images of ALP staining in hPDLSCs from different groups (Control, H_2_O_2_, ASX, H_2_O_2_ + ASX). (B) Quantitative analysis of ALP staining intensity. (C) Representative images of Alizarin Red S (ARS) staining showing mineralized nodule formation. (D) Quantitative analysis of ARS staining. (E–H) Quantitative real‐time PCR analysis of osteogenic markers RUNX2, OCN, ALP, and COL1 mRNA expression. (I) Representative Western blot images of RUNX2, OCN, ALP, and COL1 protein expression, with GAPDH as a loading control. (J–M) Semiquantitative analysis of RUNX2, OCN, ALP, and COL1 protein expression. Data are presented as mean ± SD (*n* = 3). Statistical significance:  ^∗∗^ *p* < 0.01,  ^∗∗∗^ *p* < 0.001,  ^∗∗∗∗^ *p* < 0.0001.

**Figure:**
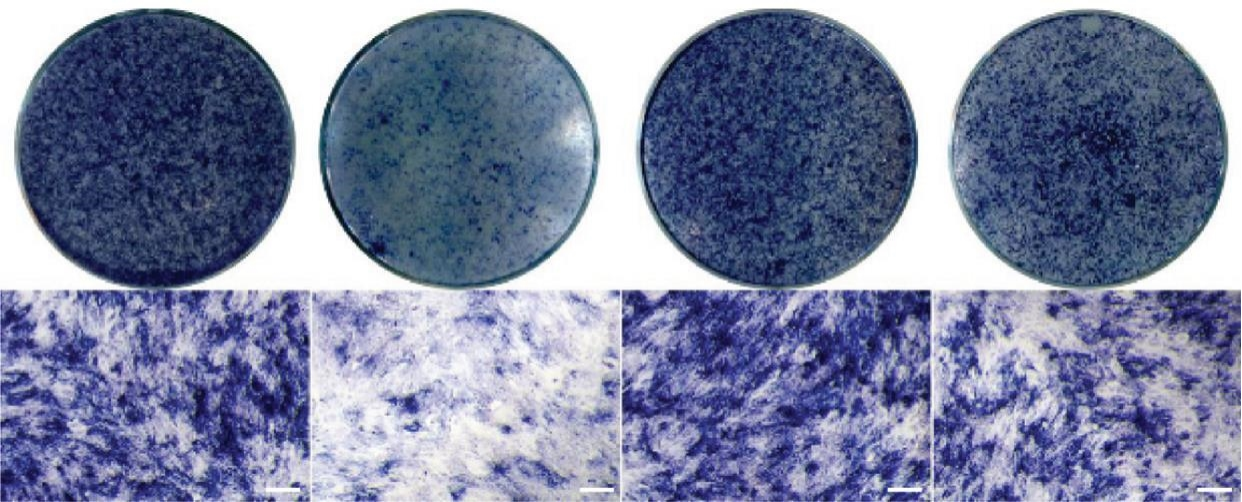
(A)

**Figure:**
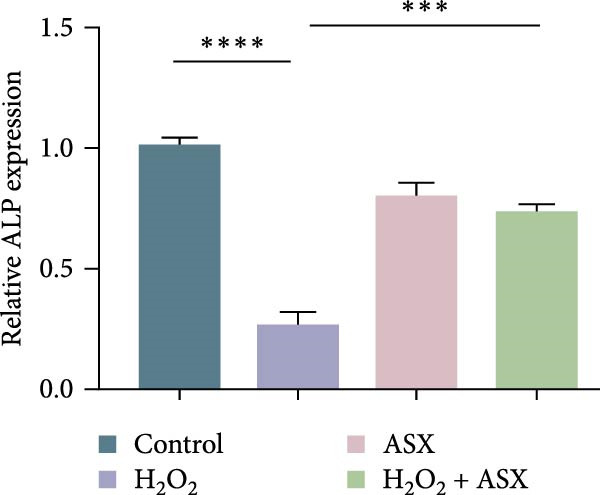
(B)

**Figure:**
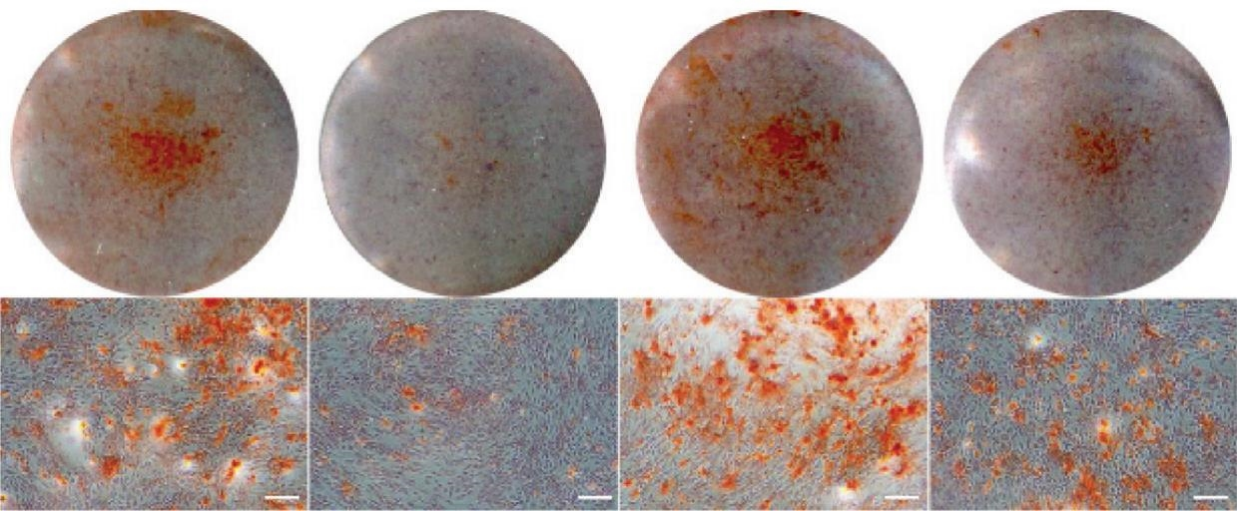
(C)

**Figure:**
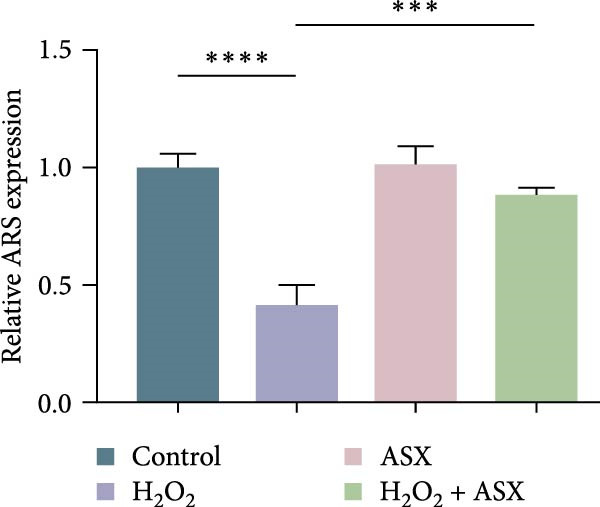
(D)

**Figure:**
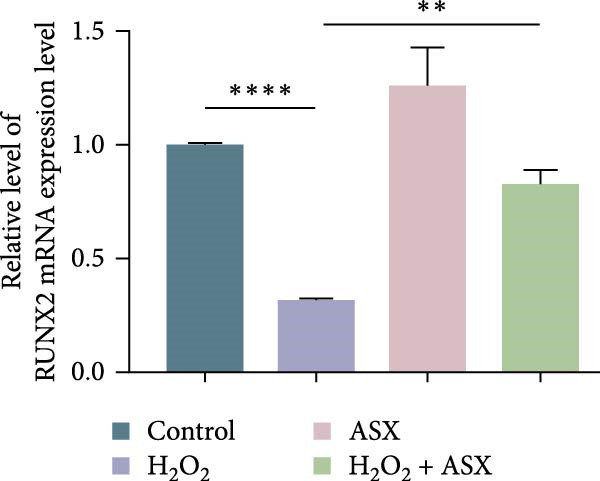
(E)

**Figure:**
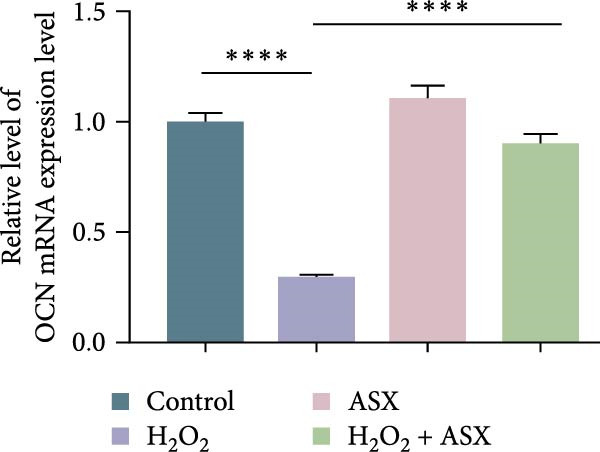
(F)

**Figure:**
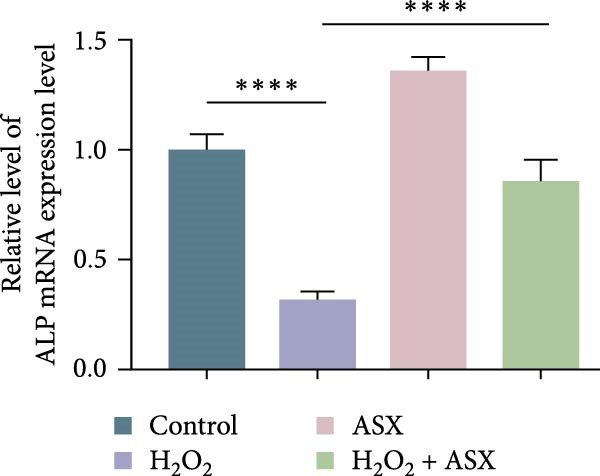
(G)

**Figure:**
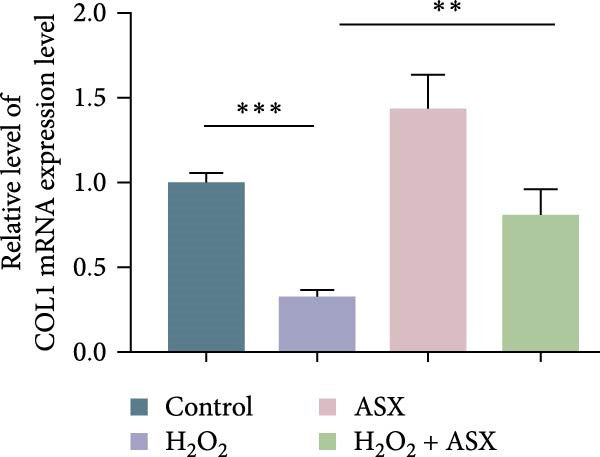
(H)

**Figure:**
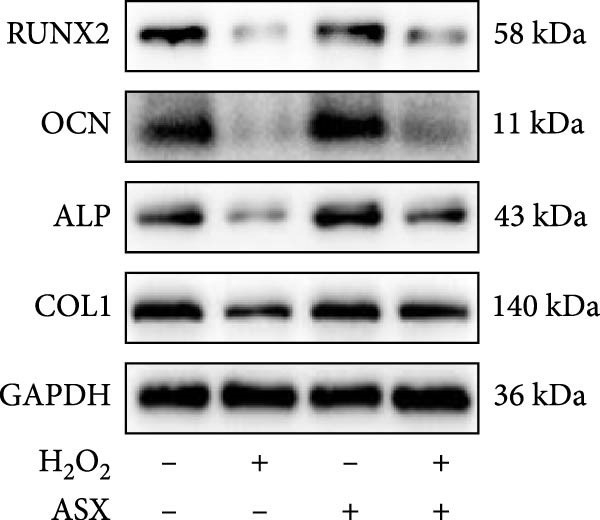
(I)

**Figure:**
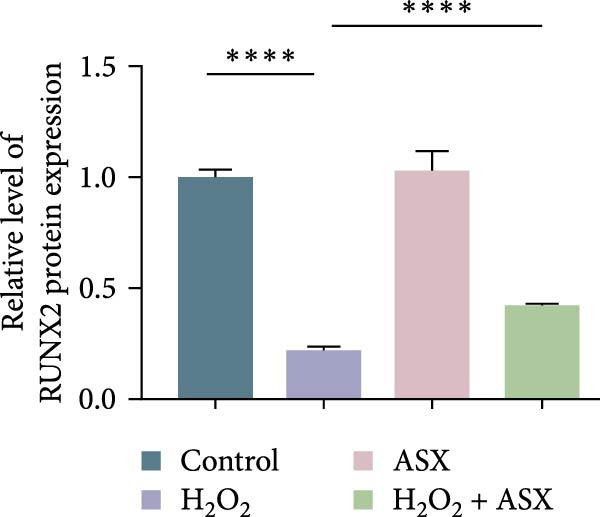
(J)

**Figure:**
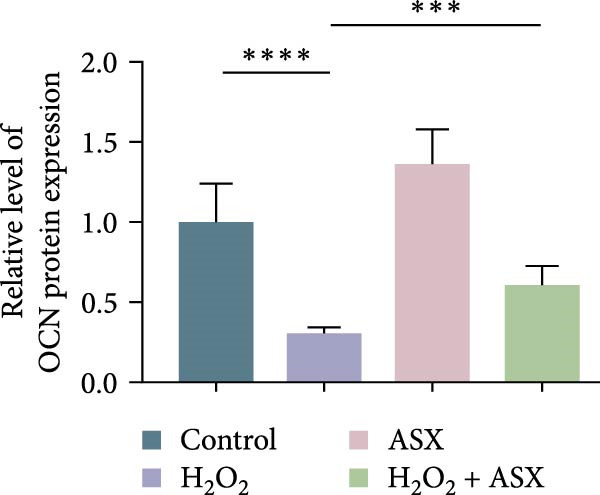
(K)

**Figure:**
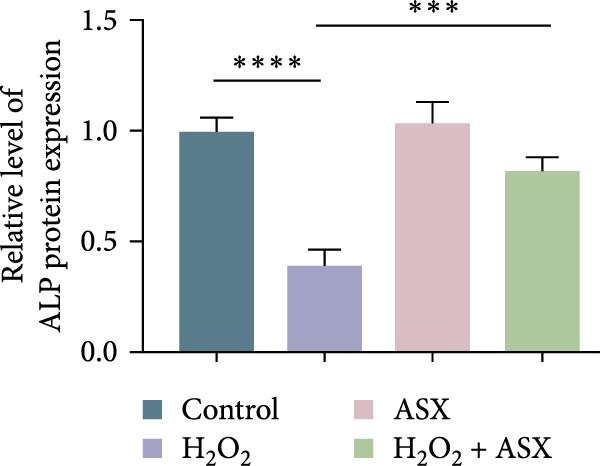
(L)

**Figure:**
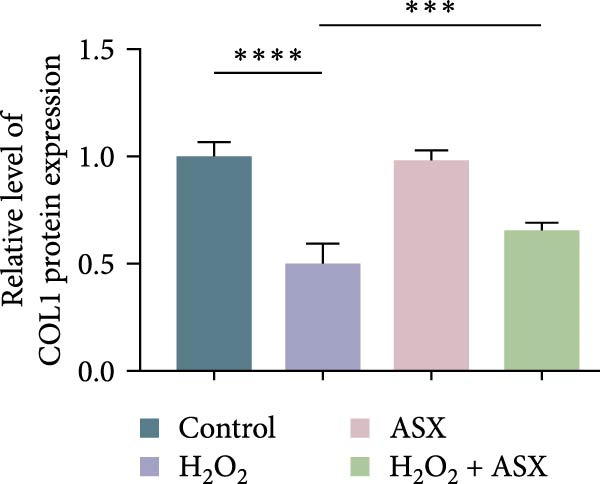
(M)

At the transcriptional level, H_2_O_2_ markedly downregulated the expression of classic osteogenic markers RUNX2, ALP, OCN, and COL1 (*p* < 0.01). ASX intervention following oxidative injury significantly restored the expression of all four genes (*p* < 0.05 vs. H_2_O_2_), approaching control levels. Protein analysis yielded consistent findings: Western blot showed reduced levels of RUNX2, OCN, ALP, and COL1 in the H_2_O_2_ group, whereas ASX‐treated cells exhibited markedly increased protein expression (*p* < 0.05). Notably, ASX treatment alone did not adversely affect osteogenesis‐related indicators, showing no significant difference from control (Figure 5E–M).

### 3.6. ASX Activates the Nrf2/ARE Signaling Pathway Under Oxidative Stress

To further elucidate the molecular mechanism underlying the cytoprotective effects of ASX, the activation status of the nuclear factor erythroid 2–related factor 2 (Nrf2)/ARE pathway was examined. qPCR results revealed that treatment with 300 μM H_2_O_2_ significantly downregulated the mRNA expression of Nrf2 and its downstream antioxidant enzymes HO‐1, NQO‐1, and GCLC compared with the control group (all *p* < 0.01, Figure 6A–D), indicating that oxidative stress suppresses the endogenous antioxidant defense system.

Figure 6ASX activates the Nrf2/ARE signaling pathway in hPDLSCs under oxidative stress. (A–D) Quantitative real‐time PCR analysis of Nrf2, HO‐1, NQO1, and GCLC mRNA expression levels in hPDLSCs from different groups (Control, H_2_O_2_, ASX, H_2_O_2_ + ASX). (E) Representative Western blot images showing the protein expression levels of Nrf2, HO‐1, NQO1, and GCLC, with GAPDH as a loading control. (F–I) Semiquantitative analysis of Western blot results for Nrf2, HO‐1, NQO1, and GCLC protein expression. Values were normalized to control ( = 100%) and are presented as mean ± SD (*n* = 3). Statistical significance:  ^∗^ *p* < 0.05,  ^∗∗^ *p* < 0.01,  ^∗∗∗^ *p* < 0.001,  ^∗∗∗∗^ *p* < 0.0001.

**Figure:**
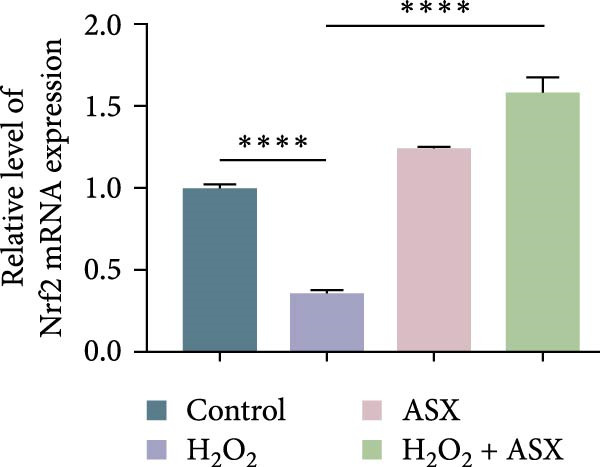
(A)

**Figure:**
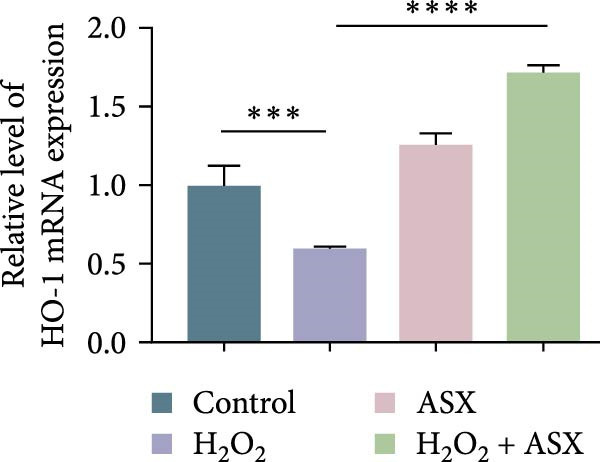
(B)

**Figure:**
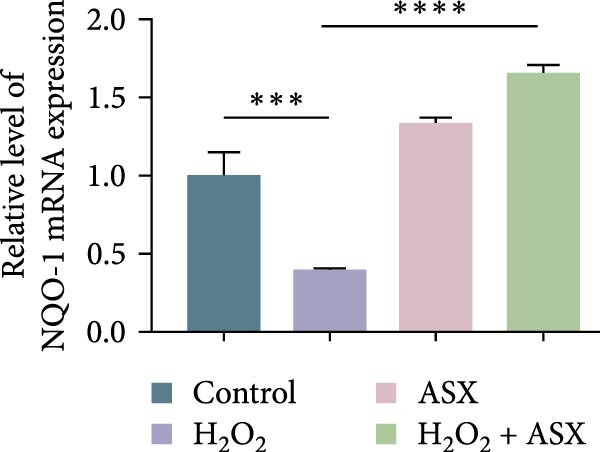
(C)

**Figure:**
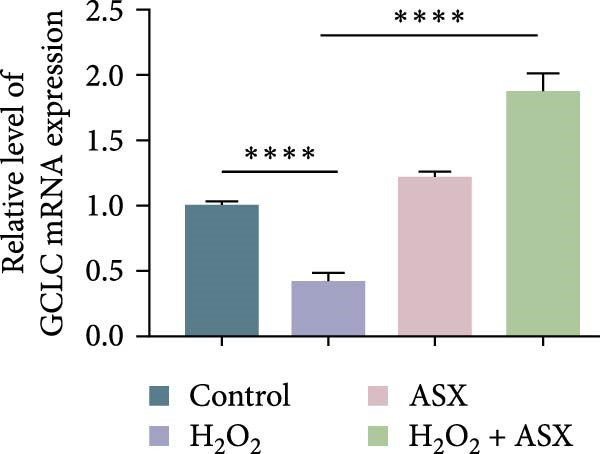
(D)

**Figure:**
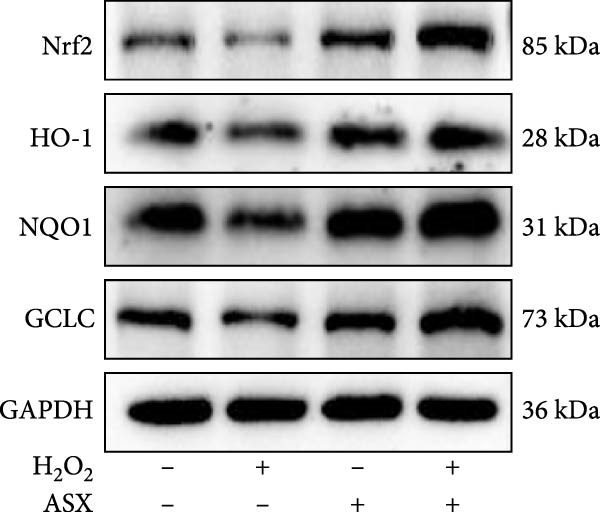
(E)

**Figure:**
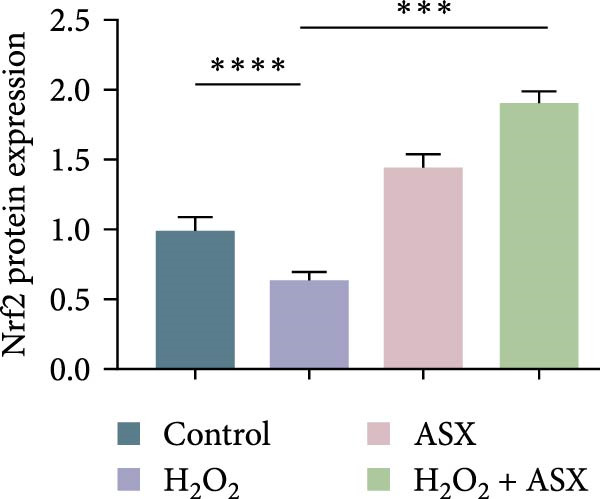
(F)

**Figure:**
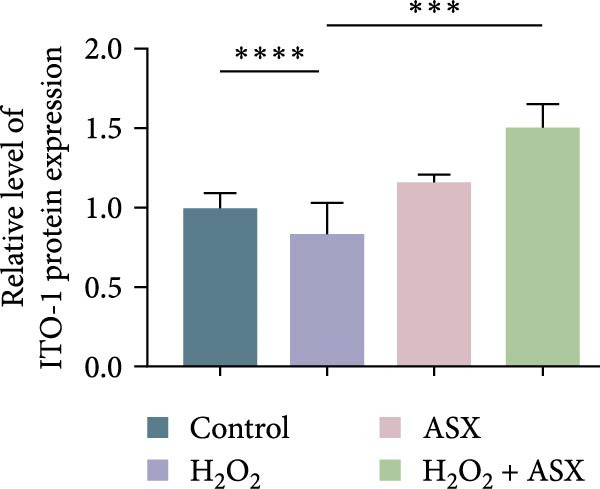
(G)

**Figure:**
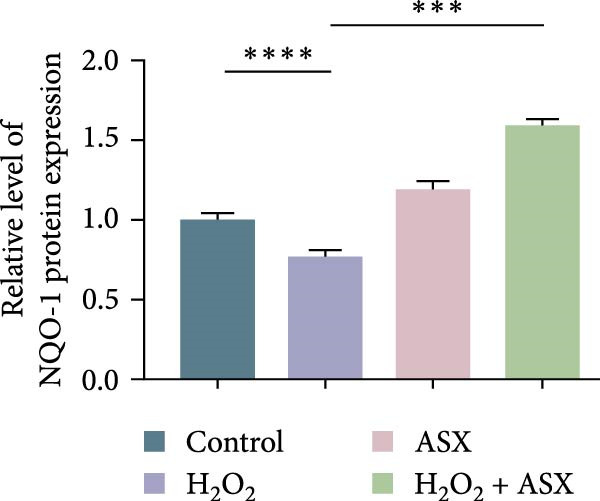
(H)

**Figure:**
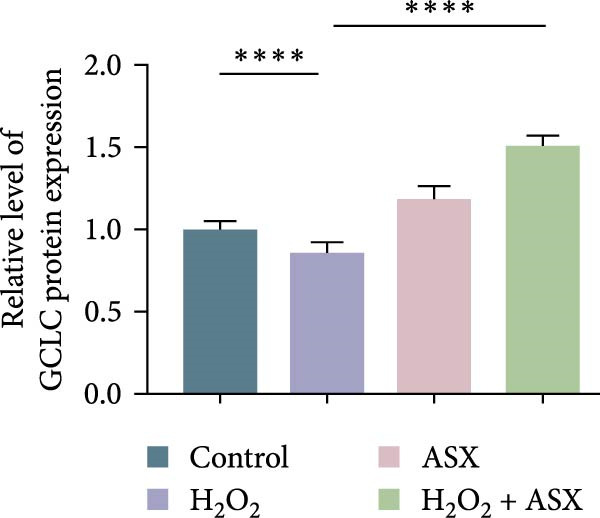
(I)

When ASX was administered following H_2_O_2_‐induced oxidative stress, the transcription levels of Nrf2, HO‐1, NQO‐1, and GCLC were significantly restored (*p* < 0.05 vs. H_2_O_2_), approaching those of the control group. Consistent with these findings, Western blot analysis showed that H_2_O_2_ markedly reduced the protein expression of Nrf2 and its downstream effectors, whereas subsequent ASX treatment substantially increased their abundance (*p* < 0.05 vs. H_2_O_2_). Densitometric quantification further confirmed that ASX effectively reactivated the Nrf2/ARE signaling pathway under oxidative stress conditions (Figure 6F–I).

### 3.7. Nrf2 Knockdown Diminishes the Antioxidant and Pro‐Osteogenic Effects of ASX

Silencing Nrf2 increased basal intracellular ROS (*p* < 0.001; Figure 7A,C). Under H_2_O_2_ challenge, siNrf2 further amplified ROS accumulation (*p* < 0.001) and reduced *ΔΨ*m (*p* < 0.05) relative to H_2_O_2_ + siNC (Figure 7A–D). ASX (10 μM) markedly lowered ROS and partially restored *ΔΨ*m compared with H_2_O_2_ alone (both *p* < 0.001), yet these benefits were attenuated by Nrf2 knockdown in H_2_O_2_ + ASX + siNrf2 (*p* < 0.05 vs. H_2_O_2_ + ASX + siNC; Figure 7A–D). Concordantly, qPCR and Western blot showed that siNrf2 decreased HO‐1, NQO1, and GCLC, and blunted ASX‐induced upregulation of these Nrf2 targets under oxidative stress (*p* < 0.05; Figure 7E–M).

Figure 7Nrf2 dependency of ASX‐mediated protection in hPDLSCs under oxidative stress. (A) Intracellular ROS detected by DCFH‐DA in control + siNC, control + siNrf2, H_2_O_2_ + siNC, H_2_O_2_ + siNrf2, H_2_O_2_ + ASX + siNC, H_2_O_2_ + ASX + siNrf2 (identical exposure/gain; scale bar, 200 μm). (B) Mitochondrial membrane potential (*ΔΨ*m) assessed by JC‐1 (red, J‐aggregates/high *ΔΨ*m; green, J‐monomers/low *ΔΨ*m). (C,D) Single‐cell quantification of intracellular ROS (C) and the JC‐1 red/green ratio (D) on background‐subtracted images using DAPI‐seeded watershed ROIs; values normalized to control ( = 100%). (E–H) qPCR of Nrf2, HO‐1, NQO1, and GCLC, normalized to GAPDH and to the Control group. (I) Representative Western blots for the above targets with GAPDH as the loading control. (J–M) Densitometric quantification of Western blots normalized to GAPDH and to Control. Values are presented as mean ± SD (*n* = 3). Statistical significance:  ^∗^ *p* < 0.05,  ^∗∗^ *p* < 0.01,  ^∗∗∗^ *p* < 0.001,  ^∗∗∗∗^ *p* < 0.0001.

**Figure:**
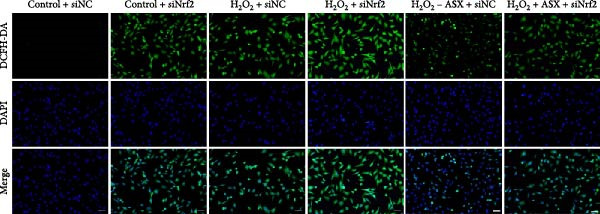
(A)

**Figure:**
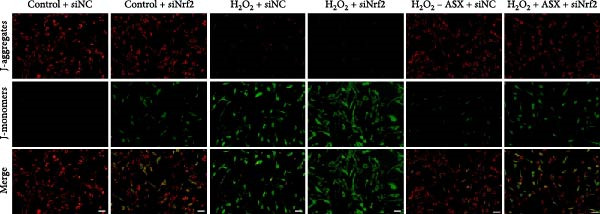
(B)

**Figure:**
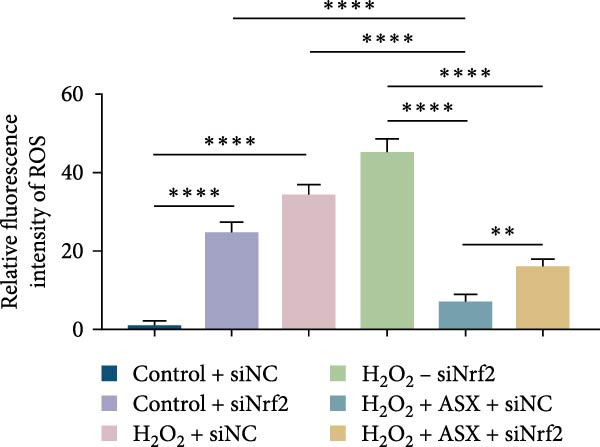
(C)

**Figure:**
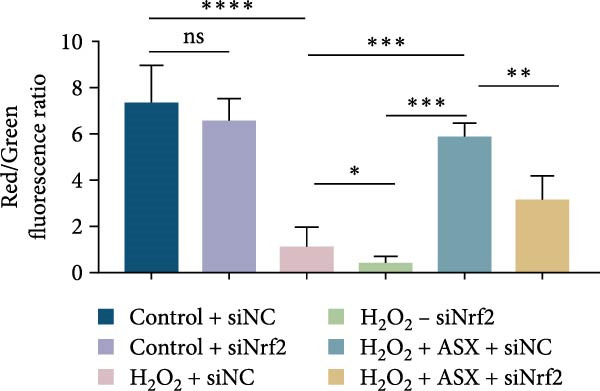
(D)

**Figure:**
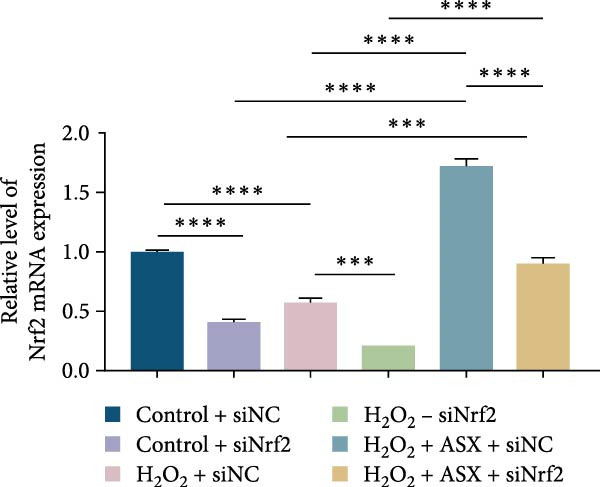
(E)

**Figure:**
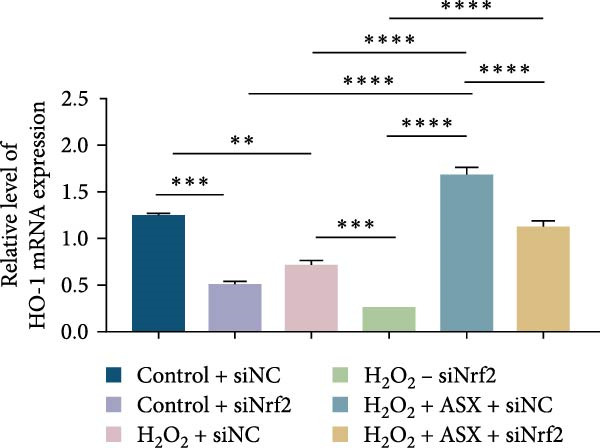
(F)

**Figure:**
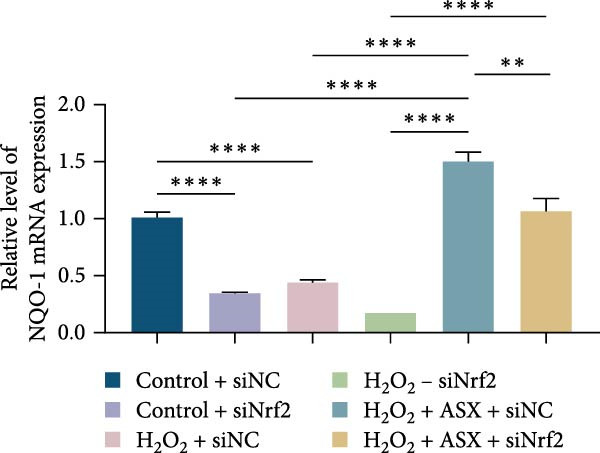
(G)

**Figure:**
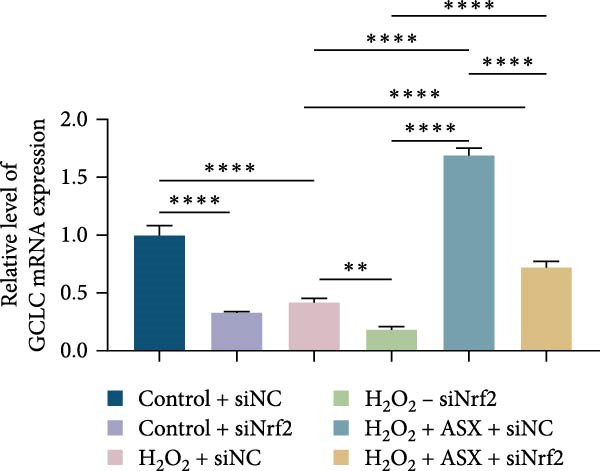
(H)

**Figure:**
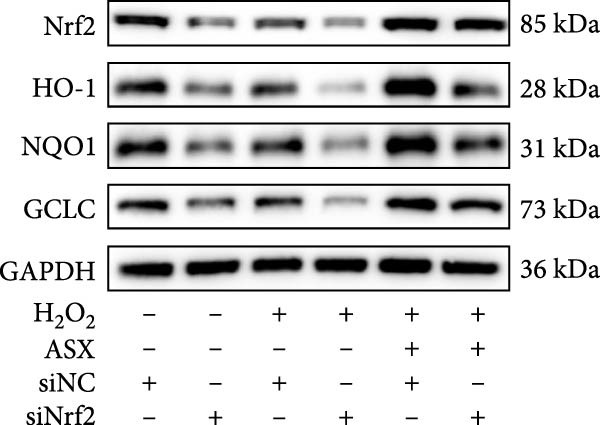
(I)

**Figure:**
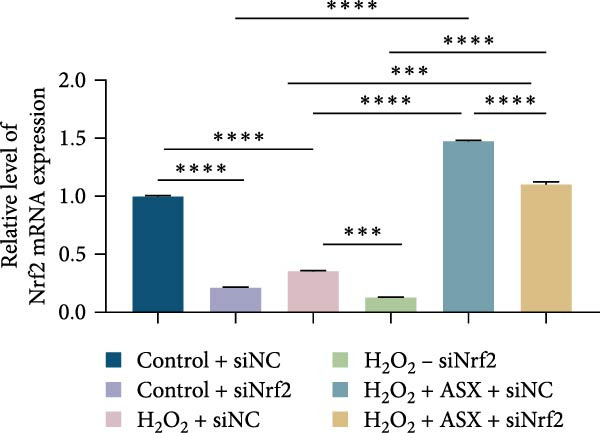
(J)

**Figure:**
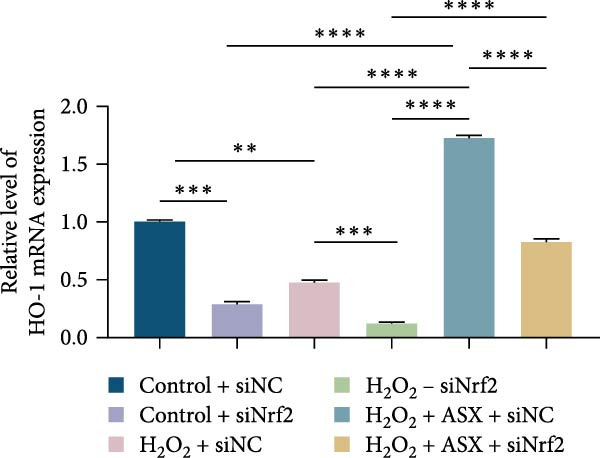
(K)

**Figure:**
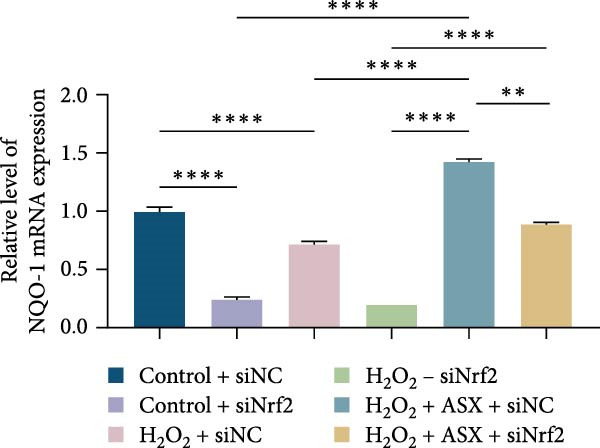
(L)

**Figure:**
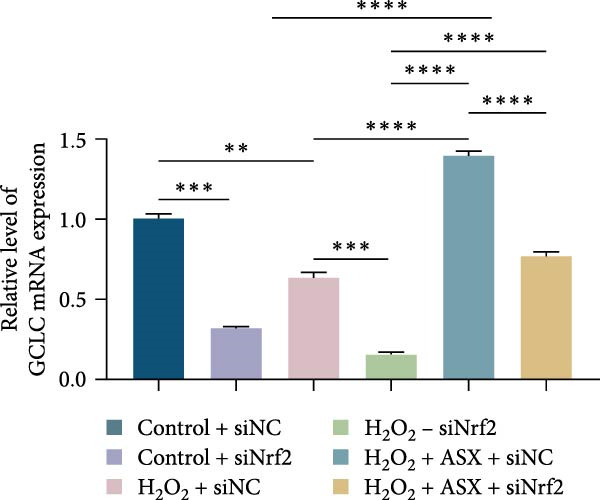
(M)

Functional outcomes mirrored these changes. H_2_O_2_ reduced ALP staining/activity and ARS‐detected mineralization (both *p* < 0.001), whereas ASX significantly improved both endpoints (*p* < 0.001 vs. H_2_O_2_; Figure 8A–D). Nrf2 knockdown lowered baseline osteogenic indices and exacerbated H_2_O_2_‐induced impairment (*p* < 0.05–0.001), and—critically—diminished the ASX‐mediated rescue (*p* < 0.05 vs. H_2_O_2_ + ASX + siNC; Figure 8A–D). At the molecular level, RUNX2, ALP, OCN, and COL1 were downregulated by H_2_O_2_ and partially restored by ASX (*p* < 0.001 vs. H_2_O_2_); this restoration was blunted by siNrf2 at both mRNA and protein levels (*p* < 0.05; qPCR: Figure 8E–H; WB/densitometry: Figure 8I–M). Collectively, these data indicate that ASX’s antioxidant, mitochondria‐preserving, and pro‐osteogenic actions in hPDLSCs require intact Nrf2 signaling.

Figure 8Astaxanthin counteracts H_2_O_2_‐induced osteogenic impairment via the Nrf2 axis. (A) ALP staining under six conditions: control + siNC, control + siNrf2, H_2_O_2_ + siNC, H_2_O_2_ + siNrf2, H_2_O_2_ + ASX + siNC, H_2_O_2_ + ASX + siNrf2 (images acquired with identical exposure/gain; scale bar, 200 μm). (B) Alizarin Red S (ARS) staining of mineralized nodules for the same six groups (scale bar, 200 μm). (C,D) Quantification of ALP (C) and ARS (D) by dye extraction/absorbance, normalized to the control group ( = 100%). (E–H) qPCR of RUNX2, OCN, ALP, and COL1, normalized to GAPDH and to Control. (I) Representative Western blots for these osteogenic proteins with GAPDH as loading control. (J–M) Densitometric quantification of Western blots, normalized to GAPDH and to Control. Values are mean ± SD (*n* = 3). Statistical significance:  ^∗^ *p* < 0.05,  ^∗∗^ *p* < 0.01,  ^∗∗∗^ *p* < 0.001,  ^∗∗∗∗^ *p* < 0.0001.

**Figure:**
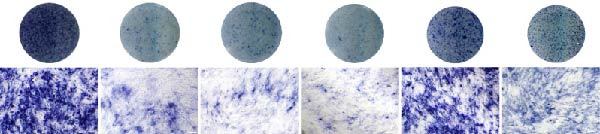
(A)

**Figure:**
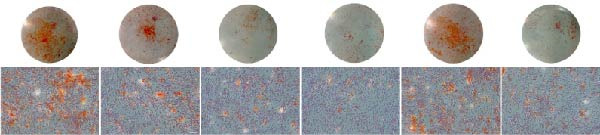
(B)

**Figure:**
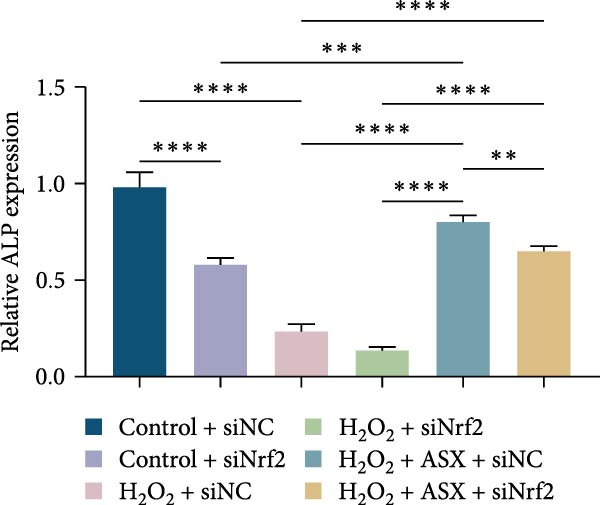
(C)

**Figure:**
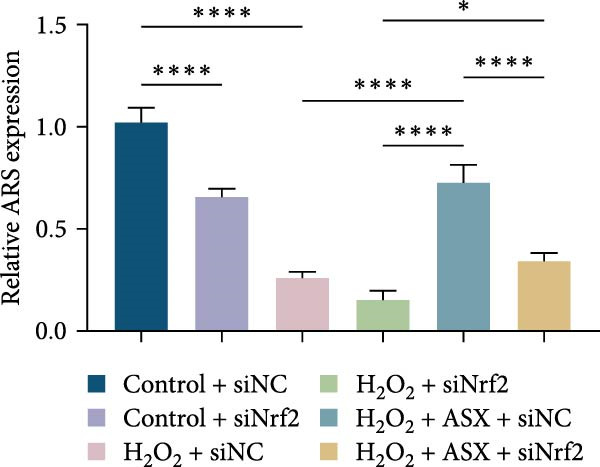
(D)

**Figure:**
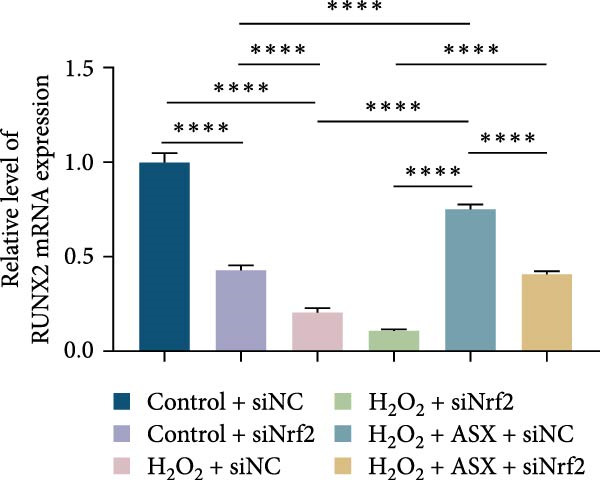
(E)

**Figure:**
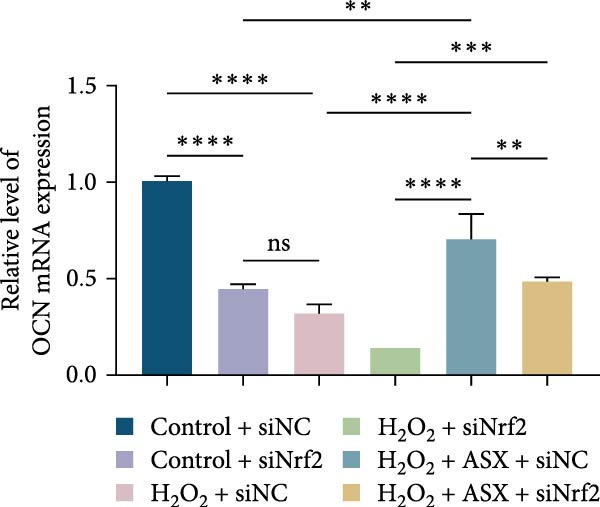
(F)

**Figure:**
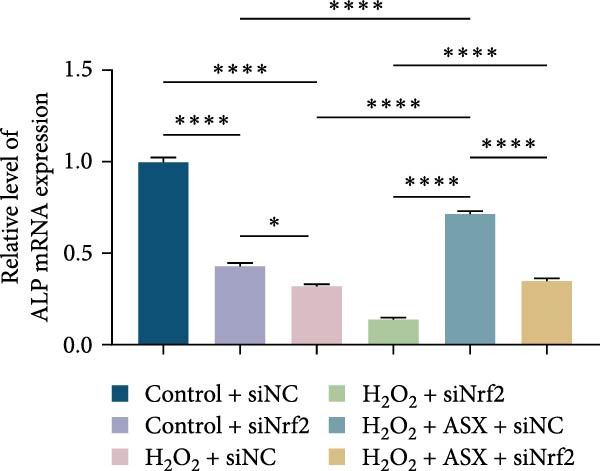
(G)

**Figure:**
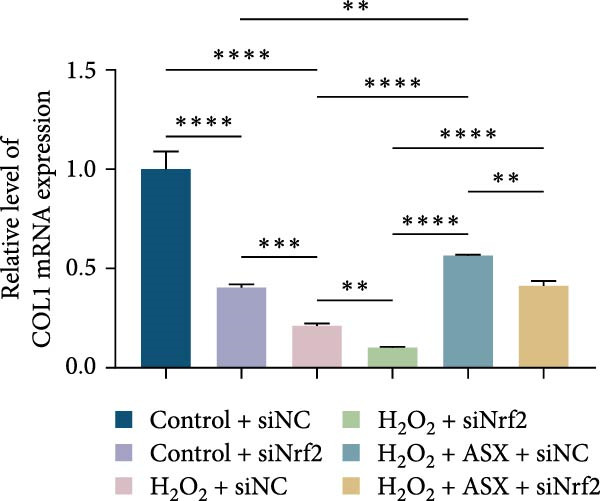
(H)

**Figure:**
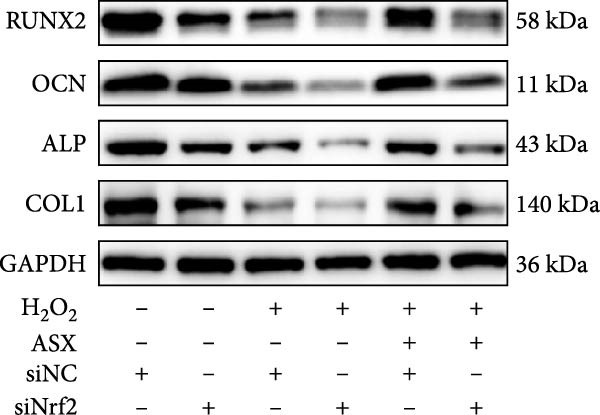
(I)

**Figure:**
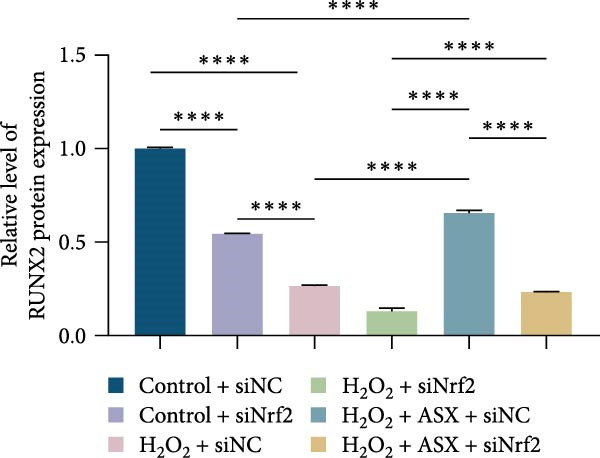
(J)

**Figure:**
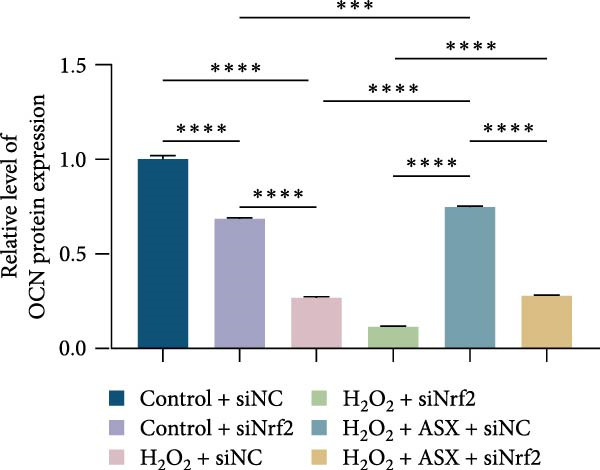
(K)

**Figure:**
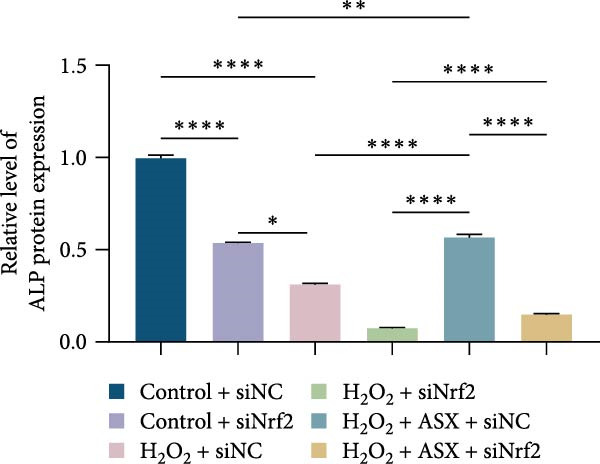
(L)

**Figure:**
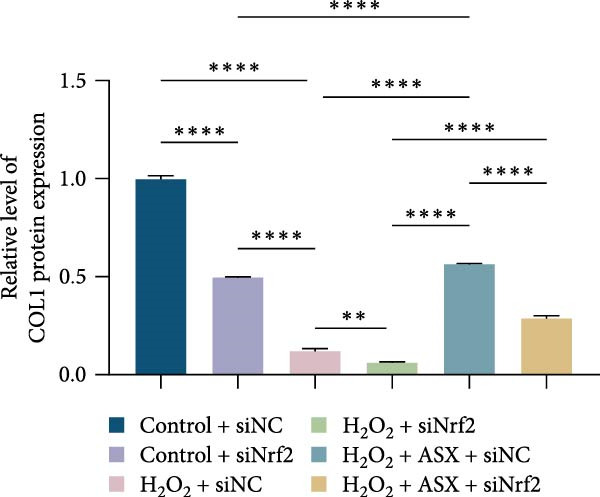
(M)

## 4. Discussion

A substantial body of evidence has demonstrated that oxidative stress plays a central role in periodontal tissue destruction. Imbalance in cellular redox homeostasis, characterized by excessive reactive ROS generation and insufficient endogenous antioxidant defense, can cause oxidative damage to DNA, proteins, and lipids, ultimately leading to cellular dysfunction and tissue degeneration [3, 18]. This pathological process highlights the potential value of enhancing cellular antioxidant capacity and maintaining redox balance in the prevention and treatment of periodontitis [19].

In this study, we confirmed that ASX, a potent lipophilic natural antioxidant, markedly alleviates hydrogen peroxide (H_2_O_2_)‐induced oxidative stress injury in hPDLSCs. ASX exerted multiple cytoprotective effects, including antioxidant, anti‐inflammatory, and pro‐osteogenic actions, which were closely associated with activation of the Nrf2/ARE signaling pathway—a key regulatory axis in maintaining cellular redox homeostasis [20].

hPDLSCs are specialized mesenchymal stem cells residing in the periodontal ligament, possessing both self‐renewal ability and osteogenic differentiation potential, making them essential seed cells for periodontal tissue regeneration [16]. Following previously reported protocols, we successfully isolated and identified hPDLSCs from healthy donor periodontal tissue. Their characteristic immunophenotype and multilineage differentiation capacity provided a reliable cellular foundation for subsequent experiments [21, 22].

To establish oxidative injury, we found that H_2_O_2_ reduced hPDLSC viability in a concentration‐ and time‐dependent manner. Treatment with 300 μM H_2_O_2_ for 6 h was identified as an optimal condition, inducing substantial oxidative stress while preserving sufficient viable cells for subsequent interventions [23–26]. ASX showed no cytotoxicity up to 16 μM, consistent with its reported biocompatibility, and 10 μM was selected as the working concentration based on safety and efficacy.

Following H_2_O_2_ induction, hPDLSCs exhibited significantly elevated cytosolic and mitochondrial ROS levels along with a marked reduction in mitochondrial membrane potential (*ΔΨ*m). These findings reflect mitochondrial dysfunction, which compromises energy metabolism and triggers apoptotic cascades [27]. Subsequent ASX treatment effectively suppressed ROS accumulation, stabilized *ΔΨ*m, and preserved mitochondrial morphology, suggesting that ASX protects mitochondrial integrity and prevents mitochondrial permeability transition pore (mPTP) opening. This observation aligns with prior reports of ASX‐mediated mitochondrial protection in models of neurodegenerative and cardiovascular injury [28, 29].

Oxidative stress is also a potent driver of inflammation [30]. In our study, H_2_O_2_ induction significantly increased mRNA and protein levels of major pro‐inflammatory cytokines (TNF‐*α*, IL‐1*β*, IL‐6, and MCP‐1), highlighting the vicious cycle between ROS and inflammation. Treatment with ASX after oxidative stress induction markedly reduced cytokine expression, indicating that its anti‐inflammatory effect results from both ROS scavenging and inhibition of pro‐inflammatory signaling. This dual mechanism suggests potential clinical utility in managing chronic inflammatory diseases such as periodontitis [31].

Regarding osteogenesis, oxidative stress impaired ALP activity, mineralized nodule formation, and expression of osteogenic markers (RUNX2, ALP, OCN, and COL1), consistent with impaired alveolar bone regeneration observed clinically [32]. Importantly, ASX treatment restored both early and late osteogenic indicators, suggesting that its osteoprotective effects extend beyond antioxidant and anti‐inflammatory actions, possibly involving activation of osteogenic pathways such as Wnt/*β*‐catenin, as reported in other regenerative contexts [33, 34].

At the mechanistic level, we confirmed that the Nrf2/ARE pathway mediates ASX’s cytoprotective actions. H_2_O_2_ exposure suppressed Nrf2 and its downstream antioxidant enzymes (HO‐1, NQO1, and GCLC), thereby weakening endogenous antioxidant defenses, whereas ASX reversed this suppression, promoting Nrf2 activation and transcription of ARE‐dependent genes [35, 36]. Notably, Nrf2 and its downstream genes were expressed at higher levels in the H_2_O_2_ + ASX condition than in either Control or ASX alone. We posit that during the early phase of H_2_O_2_‐induced oxidative stress, oxidation of critical Keap1 cysteines weakens Keap1‐mediated ubiquitination of Nrf2, releasing repression and producing a transient rise in Nrf2 activity; when the insult becomes excessive and sustained, however, mitochondrial dysfunction and depletion of reducing equivalents (e.g., GSH/NADPH), together with global constraints on transcription/translation, drive a decay of Nrf2 signaling. By lowering excessive ROS, preserving mitochondrial function and cellular reducing capacity, and potentially engaging pro‐survival kinases (e.g., PI3K/Akt, AMPK/MAPK), ASX prolongs the nuclear retention and transcriptional competence of Nrf2; accordingly, Nrf2/ARE readouts in H_2_O_2_ + ASX exceed those in Control or ASX alone. Consistent with this mechanism, siNrf2 increased basal ROS, exacerbated H_2_O_2_‐induced ROS accumulation and loss of *ΔΨ*m, and blunted ASX‐driven upregulation of HO‐1, NQO1, and GCLC and the recovery of RUNX2, ALP, OCN, and COL1, underscoring the pathway dependency of ASX’s effects. Since Nrf2 not only regulates ROS clearance but also modulates inflammation and osteogenesis, its reactivation—and the loss‐of‐function evidence above—provides a coherent mechanistic basis for the multi‐faceted protective effects of ASX.

From a translational standpoint, effective periodontitis therapy requires simultaneous control of inflammation and promotion of tissue regeneration, with oxidative stress serving as a common mechanistic link. ASX, with its combined antioxidant, anti‐inflammatory, and pro‐osteogenic properties, represents a promising candidate for adjunctive periodontal regenerative therapy. Practical considerations include local delivery to periodontal pockets (e.g., hydrogel or microparticle systems) to achieve micromolar tissue levels while minimizing systemic exposure, dosing aligned with debridement/regenerative procedures, and prioritization of patients with heightened oxidative burden (e.g., smokers and diabetes). Nonetheless, this study was conducted in vitro and cannot fully replicate the complexity of the periodontal microenvironment, which includes bacterial biofilms, immune cell interactions, and mechanical forces. Future work should extend to chronic, biofilm‐relevant oxidative models, incorporate gain‐of‐function (Nrf2 overexpression) to complement knockdown, and evaluate pharmacokinetics, target engagement, and functional outcomes (bone fill and PDL regeneration) in vivo.

In conclusion, ASX mitigates H_2_O_2_‐induced oxidative injury in hPDLSCs by activating the Nrf2/ARE pathway, reducing ROS accumulation, preserving mitochondrial function, suppressing inflammation, and restoring osteogenic capacity. These findings deepen our understanding of ASX’s multifunctional bioactivities and provide a theoretical foundation for its therapeutic potential in oxidative stress‐related periodontal diseases.

## 5. Conclusion

This study demonstrates that ASX alleviates oxidative stress–induced injury in hPDLSCs by activating the Nrf2/ARE pathway, thereby reducing ROS accumulation, preserving mitochondrial function, suppressing inflammation, and restoring osteogenic capacity. These antioxidant, anti‐inflammatory, and pro‐osteogenic effects highlight ASX as a promising adjunctive strategy for managing oxidative stress–related periodontal damage.

## Consent

Informed consent was obtained from all subjects involved in the study.

## Disclosure

No copyrighted material from other sources was used in this study.

## Conflicts of Interest

The authors declare no conflicts of interest.

## Funding

This research did not receive any specific grant from funding agencies in the public, commercial, or not‐for‐profit sectors.

## Data Availability

The datasets generated and/or analyzed during the current study are available from the corresponding author upon reasonable request.
